# Comparative Genome Sequencing Reveals Within-Host Genetic Changes in *Neisseria meningitidis* during Invasive Disease

**DOI:** 10.1371/journal.pone.0169892

**Published:** 2017-01-12

**Authors:** Johanna Klughammer, Marcus Dittrich, Jochen Blom, Vera Mitesser, Ulrich Vogel, Matthias Frosch, Alexander Goesmann, Tobias Müller, Christoph Schoen

**Affiliations:** 1 CeMM Research Center for Molecular Medicine of the Austrian Academy of Sciences, Vienna, Austria; 2 Institute for Hygiene and Microbiology, University of Würzburg, Würzburg, Germany; 3 Department of Bioinformatics, Biocenter, University of Würzburg, Würzburg, Germany; 4 Institute of Human Genetics, Biocenter, University of Würzburg, Würzburg, Germany; 5 Institute for Bioinformatics and Systems Biology, Justus Liebig University Giessen, Giessen, Germany; 6 Research Center for Infectious Diseases, University of Würzburg, Würzburg, Germany; 7 German Reference Laboratory for Meningococci and Haemophilus influenzae, Institute for Hygiene and Microbiology, University of Würzburg, Würzburg, Germany; Universidad Nacional de la Plata, ARGENTINA

## Abstract

Some members of the physiological human microbiome occasionally cause life-threatening disease even in immunocompetent individuals. A prime example of such a commensal pathogen is *Neisseria meningitidis*, which normally resides in the human nasopharynx but is also a leading cause of sepsis and epidemic meningitis. Using *N*. *meningitidis* as model organism, we tested the hypothesis that virulence of commensal pathogens is a consequence of within host evolution and selection of invasive variants due to mutations at contingency genes, a mechanism called phase variation. In line with the hypothesis that phase variation evolved as an adaptation to colonize diverse hosts, computational comparisons of all 27 to date completely sequenced and annotated meningococcal genomes retrieved from public databases showed that contingency genes are indeed enriched for genes involved in host interactions. To assess within-host genetic changes in meningococci, we further used ultra-deep whole-genome sequencing of throat-blood strain pairs isolated from four patients suffering from invasive meningococcal disease. We detected up to three mutations per strain pair, affecting predominantly contingency genes involved in type IV pilus biogenesis. However, there was not a single (set) of mutation(s) that could invariably be found in all four pairs of strains. Phenotypic assays further showed that these genetic changes were generally not associated with increased serum resistance, higher fitness in human blood *ex vivo* or differences in the interaction with human epithelial and endothelial cells *in vitro*. In conclusion, we hypothesize that virulence of meningococci results from accidental emergence of invasive variants during carriage and without within host evolution of invasive phenotypes during disease progression *in vivo*.

## Introduction

The importance of the human microbiome in health and disease has received increasing attention in recent years [1]. Although these microbial populations are normally beneficial or neutral, some components of these populations, called commensal pathogens, can cause disease and are responsible for most human bacterial infections. Prominent examples include *Haemophilus influenzae*, *Streptococcus pneumoniae*, and *Neisseria meningitidis*, which colonize the upper respiratory tract of humans as harmless commensals but are also leading causes of acute bacterial meningitis and septicemia [2].

*N*. *meningitidis* is one of the few commensal bacteria that can even cause large epidemics of invasive disease [3]. However, invasive meningococcal disease (IMD) is an evolutionary dead end for these species, because bacteria residing in the bloodstream are rarely transmitted to new hosts. Consequently, also nominated “virulence factors” in these bacteria must have evolved in response to selection for something other than to cause invasive disease.

Although certain host-genetic factors and adaptive immunity are associated with an increased susceptibility to IMD [4], there is so far no evidence that genetic predisposition of the human host is a major requirement for the development of IMD. In turn, molecular epidemiology has provided clear observational evidence for a significant association between certain bacterial genotypes (hyperinvasive lineages) and IMD [5]. However, genomic comparison of hyperinvasive and apathogenic lineages did not reveal unambiguous evidence of the presence of indispensable virulence factors [6, 7].

The hypothesis of within-host evolution (WHE) posits that virulence (host damage) in commensal pathogens evolves within individual hosts and without regard to the ultimate survival, i.e. transmission, of the pathogen in the host population [8–10]. It states that virulence is a consequence of shortsighted within-host competition among genetically diverse subpopulations, particularly due to phase variation at contingency loci [11]. Phase variation occurs randomly mainly via slipped-stranded mispairing (SSM) at simple sequence repeats (SSRs) during replication and often affects genes involved in host interactions [11, 12]. Epidemiological models suggest that phase variation evolved as an adaptation for colonization of diverse hosts [10, 13].

Under the assumption of direct selection during WHE, virulence is correlated with bacterial fitness within the host. Accordingly, mutants that are randomly generated in the nasopharynx from colonizing ancestors cross the nasopharyngeal barrier and are selected for better survival in the bloodstream thus successfully populating an empty ecological niche within the host [9, 10]. Being the result of coincidental selection, virulence occurs due to selection for other functions and does not confer any advantage in the infected host such as survival in the bloodstream [8, 14]. Here, virulence is not correlated with any measure of bacterial success such as within-host growth advantage, and host damage results from “accidental” bacteria-host interaction.

Although first experimental evidence for WHE was obtained in an infant rat model of *H*. *influenzae* bacteremia [15], the recent development of powerful next-generation sequencing (NGS) technologies has opened new avenues for the experimental study of bacterial within-host genomic changes even during acute infections [16–18]. Obtaining reliable sequencing results particularly at SSR loci is paramount, but analysis remains challenging due to high error rates inherent to certain types of NGS data [16, 19].

Here, we used *N*. *meningitidis* as a model to test the WHE hypothesis using throat-blood isolate pairs from four patients suffering from acute IMD. We combined comparative genomics, different sequencing technologies, and scrupulous bioinformatics data processing to assess genomic changes with base pair accuracy and finally tested the fitness of the respective strains in human cell culture and *ex vivo* assays. We show that within-host genomic changes occur frequently in meningococci during IMD, and while there was some association of these mutations with the biogenesis and function of the meningococcal type four pilus (Tfp) we could not identify a single (set) of mutation(s) that could invariably be found in all four strains pairs. We further found small yet statistically non-significant differences in some strain pairs in resistance against human serum as well as in adhesion to and invasion of human epithelial cells, whereas meningococcal fitness in the bloodstream was not affected. We hypothesize that virulence of meningococci results from accidental evolution of invasive variants during carriage and without within host selection of invasive phenotypes during disease. Despite the limited number of strain-pairs this first pilot study of meningococcal within-host evolution provides an important resource for the design and implementation of larger multi-center studies.

## Materials and Methods

### Bacterial strains

Upon receipt at the NRZMHi, all samples are routinely streaked out on Martin-Lewis agar plates (Becton Dickinson, Heidelberg, Germany) and incubated overnight at 35°C in 5% CO_2_. The next day, a sweep collecting all colonies grown on the agar plate was taken and frozen at -70°C in standard I nutrient broth (25 g/l) (Merck Millipore, Darmstadt, Germany) supplemented with 20% glycerol without further processing. For further analyses, meningococcal strains were grown on Columbia blood agar plates (bioMérieux, Nürtingen, Germany) directly from the stocks without further passages. The eight strains examined in this study are described in Table 1.

**Table 1:** Overview over the patient and the respective isolates.

	Patient 1	Patient 2	Patient 3	Patient 4
Age (years)	2	24	16	17
Sex	f	m	f	m
Year	2002	2002	2005	1997
Disease	WFS*	WFS*	Sepsis	Sepsis
Throat isolate	DE8555	DE8669	DE10444	WUE2121
Blood isolate	DE8539	DE8678	DE10445	WUE2120
Serogroup	C	B	Y	C
Sequence Type	ST-11	ST-42	ST-23	ST-11
Genome size [bp]	2,207,969	2,230,062	2,170,563	2,206,831

*Waterhouse-Friderichsen Syndrome

### De novo sequencing and assembly of throat isolate genomes

Chromosomal DNA was isolated from bacteria grown in 5 ml of Proteose Peptone Medium supplemented with 1% Polyvitex (bioMérieux) using QIAGEN Genomic-tip 20/G (Qiagen, Hilden, Germany). Genomic DNA from the four throat isolates (DE10444, DE8669, WUE2121, DE8555) was sequenced *de novo* by Eurofins MWG Operon, (Ebersberg, Germany) using the next generation sequencing platform 454 Genome Sequencer FLX (GS FLX) from Roche. The data sets were generated from large paired-end libraries (LPE; with estimated 8 kbp span distance) and whole-genome shotgun libraries (SG). Contigs were generated using a Celera assembler version 6.2 based automatic assembly pipeline (in-house software, Eurofins MWG Operon). Sequencing statistics are displayed in S1 Table.

Using the scaffolding information derived from the sequencing data of the four GS FLX LPE libraries, primer pairs were designed and synthesized for a PCR-based approach of gap closure. The PCR-products were sequenced by capillary electrophoresis employing the dye terminator chemistry (Applied Biosystems 3730XL DNA Analyzer). Due to the structure characteristics of the DNA, a PCR-kit for GC-rich DNA had to be used for amplicon generation and dGTP chemistry for sequencing. The results of the Sanger sequencing were co-assembled with the GS FLX data in an iterative approach. In order to close the remaining gaps the Basic Local Alignment Search Tool (BLAST) was used to identify possible overlaps between contigs. Thereafter, the joining of the contigs was performed manually using Staden Package Gap4 (http://staden.sourceforge.net/). The mapping of intermediate consensus-sequences of the contigs against the given reference sequences (DE8555: FAM18; DE8669: α710; DE10444: Z2491; WUE2121: FAM18) were performed for the same purpose. The final consensus sequence was verified and improved by an additional mapping using MIRA3 software (version 3.2.1; http://mira-assembler.sourceforge.net/). Through this concerted approach high-quality consensus sequences could be generated for all four isolates.

### Whole-genome re-sequencing of the blood and throat isolate genomes

Whole genome sequencing libraries of all eight isolates (Table 1) were prepared using the TruSeq DNA Sample Preparation Protocol/Kit v2, producing libraries with an estimated insert size of ~300 bp. Each sample was barcoded with a unique and compatible index sequence to enable equimolar multiplexing of all eight samples. The multiplexed library was sequenced by the Biomedical Sequencing Facility at CeMM in one lane on the Illumina HiSeq 2000 platform using the 100 bp paired-end configuration and Illumina v3 reagents. This resulted in over 31 million reads per sample and an average coverage of ~ 1500-fold. Demultiplexing, quality filtering, and conversion to fastq file format was performed using the Illumina/CASAVA pipeline (v1.8.2). Sequencing statistics are displayed in S1 Table.

### Read mapping and variant calling

Bowtie (v2.0.2) [20] was run in the --very-sensitive and --end-to-end mode with the expected insert size set to 250 to 500 bps. Variants were called by combining samtools and bcftools [21]. Samtools mpileup (v0.1.18-r580) was run with the following command: *samtools mpileup -L8000 -uf $reference $sorted_bam > $pileup*. Variants were then extracted from the pileup file using the following command: *bcftools view -Svcg $pileup > $raw_variants*.

### Variant filtering

For each strain the detected variants were classified into two categories: Variants detected in either the throat or the blood isolate (unique variants) and variants detected in the throat as well as the blood isolate (common variants). While the unique variants represent potential genomic differences between throat and blood isolates, the common variants represent potential sequencing and/or assembly errors in the reference genome. In order to reduce false positives and focus on variants with a likely biological relevance, we devised a variant filtering strategy that takes into account quality (quality filter) and effect (effect filter) of a variant. The filtering criteria were specific to each variant class and chosen to be selective for common variants (low false positive rate) and sensitive for unique variants (low false negative rate): In order to be accepted as a true common variant, the ratio of reads supporting the reference to reads supporting the variant must be smaller than or equal to 0.25 in both isolates (quality filter) and the variant must be called unambiguously (effect filter). The cutoff ratio of 0.25 has been arbitrarily set to a very stringent value in order to keep the false positive rate low. Likewise, in order to be accepted as a true unique variant, single nucleotide variants (SNVs) need be assigned a phred-scaled quality score of greater than or equal to 100 by the variant caller while this quality filter was not applied on small insertions and deletions (indels) due to the inherent difficulty of calling indels accurately and with high confidence. This threshold was chosen based on the smoothed (method: loess, confidence interval 0.95) occurrence distribution of variant quality scores (S1 Fig). The common variants can be seen as likely to be true variants in a technical- but not biological sense since they have been called independently in two samples. Therefore, the quality score threshold of 100 was chosen as the value at which the occurrence distribution for the common variants significantly rises above zero. Furthermore, unique variants are required to lie within the CDS or the promoter (up to 100 bp upstream of the CDS) of a gene (effect filter) in order to be considered for validation by Sanger sequencing (S2 Table).

### Annotation and correction of throat isolate genome assemblies

To correct for Roche-454 specific sequencing errors in the newly assembled reference genomes that had been identified by Illumina resequencing (common variants as described above), a semi-automated approach was chosen. First a correction matrix specifying genomic position and variant was manually created, then based on this matrix a custom R script (based on Bioconductor/Biostrings [22]) was used to apply the specified changes to the reference genome sequence.

Automatic genome annotation was performed using the GenDB platform [23]. Region prediction was realized using Prodigal [24] for coding sequences (CDS), tRNAscan-SE [25] for tRNAs, and RNAMMER [26] for rRNAs. Functional annotation was based on consecutive comparisons against public sequence databases: Uniprot/Siwssprot [27], NCBI databases Refseq and NR [28], Pfam-A [29] and TIGRFAMS [30]. Finally, TMHMM [31], helix-turn-helix [32] and SignalP [33] were used on all annotated CDS for function and sub-cellular localization prediction for all encoded proteins.

### Accession numbers of the throat isolate genomes

The GenBank accession numbers for the whole-genome sequences of the four throat isolates are given in S3 Table. The Illumina resequencing data for throat and blood isolates have been submitted to SRA and are available under the same accession.

### Simple Sequence Repeat (SSR) locus identification

Simple sequence repeats up to a unit length of 10 bases were identified using the command line version of Tandem Repeats Finder (TRF v.4.04) [34] with the following parameter settings:

*Match = 4 (matching weight)*, *Mismatch = 16 (mismatching penalty)*, *Delta = 16 (indel penalty)*, *PM = 80 (match probability)*, *PI = 10 (indel probability)*, *Minscore = 23 (minimum alignment score to report)*, *MaxPeriod = 10 (maximum period size to report)*. These parameters were chosen to not allow any mismatchs within the repeat (high penalty values) and at the same time require a minimum of 6 matches because SSRs of total length < 6 are not considered relevant as described below.

Candidate SSR loci were identified based on the following criteria adapted from Saunders *et al*. [35]: The repeat must be located within a coding sequence (CDS) or at most 100 bp upstream of the CDS. G and C homopolymers must have a length of greater than 6 bp. A and T homopolymers must have a length of greater than 8 bp. GC/CG dinucleotide repeats must consist of more than 4 repeats; all other dinucleotide or trinucleotide repeats must consist of more than 3 repeats. Tetranucleotide repeats to repeats with a maximum period length of 10 nucleotides (decanucleotide repeats) must consist of more than 2 repeats. Only repeats with 100% identity were considered.

In contrast to the criteria defined by Saunders *et al*. [35], trinucleotide repeats and multiples thereof were not excluded from further analysis because different repeat lengths might influence gene expression despite the fact that trinucleotide deletions (and multiples thereof) do not lead to frameshifts when located within CDSs.

### Determining the core set of SSR loci in 27 complete meningococcal genomes

In order to determine orthologous SSR loci within the SSR loci identified in 23 published [6, 36–45] as well as the four newly sequenced *N*. *meningitidis* throat isolate genomes (S3 Table) all-against-all nblast [46] was performed using CDS fasta files as input and setting the identity threshold to 90%. Additionally, at least 90% of the query sequence needed to align to the subject sequence in order to be considered a true match. SSR loci were grouped into clusters of high sequence similarity based on the blastn bitscore [46, 47] using the dist, hclust and cutree functions of the R package stats [48]. The most suitable distance measure (“binary”), agglomeration method (“mcquitty”) and number of clusters (621) were chosen to optimize the following three conditions: (i) Minimize the total number of clusters while limiting the number of genes per cluster to not excessively exceed the total number of assessed genomes (except for paralogous genes which can lead to more than one gene per genome and cluster). (*ii*) Maximize the number of clusters that contain at least one gene of each of the assessed genomes. (*iii*) Minimize the number of clusters representing only one gene. The quality of the clusters was checked by performing multiple sequence alignments with all genes grouped into one cluster (data not shown). Cluster cohesion was estimated by calculating the length corrected mean bitscore for each cluster. The alignments for least cohesive core-clusters were assessed visually and approved. Based on this ortholog clustering approach the *N*. *meningitidis* “core SSR loci” were defined as those clusters that contained at least one ortholog from every assessed genome, while the “pan SSR loci” consisted of all of the 621 different orthologous SSR locus clusters. In addition, the relation between the number of assessed genomes to the number of genes identified as “core” and “pan” was assessed by calculating the number of genes present in all assessed strains (“core”) and the total number of different genes present in any of the assessed genomes (“pan”) for each possible combination of one to 27 genomes. This relation was then fitted by a power law function [49] to the calculated medians using the nls function of the R package stats. The power law function was defined as a * i^b^ + c. For the description of the “pan SSR loci” the starting values were set to (a = 100, b = 0.4, c = -100). For the description of the “core SSR loci” the starting values were set to (a = 100, b = -0.6, c = 100). Confidence intervals, to a confidence level of 0.95, were computed from the fitted models using the function confint.

### Cluster of Orthologous Groups (COG) enrichment analysis

We used gene set enrichment analysis to analyze the distribution of genes identified as SSR loci over the different COG functional classes [50] and to assess whether potentially phase-variable genes code for certain functions more frequently than expected by chance. As this approach requires whole-genome sequences, we consequently retrieved only fully sequenced, assembled, and annotated meningococcal genomes from the NCBI public database, totaling 27 at the time of analysis (May 2016). Genes of all assessed *N*. *meningitidis* genomes were further functionally annotated using the COGnitor framework provided by MicroScope (http://www.genoscope.cns.fr), and gene set enrichment analysis was performed using the Fisher’s exact test together with the Benjamini-Hochberg correction for multiple testing [51].

### Whole-genome alignments of ST-11 genomes

The progressiveMauve algorithm as implemented in the Mauve software package [52] was used for whole-genome alignments of strains from the ST-11 clonal complex (S3 Table). Single nucleotide variants were assessed using the “Export SNP” option in pairwise comparisons.

### Probability estimations for Tfp contingency genes

According to a recent genome-wide transposon mutagenesis study [41], about 23 genes are involved in Tfp function, of which *K* = 7 are known to be phase-variable contingency genes [36, 41]. Based on our genome-wide analysis, we further assume up to *n* = 3 (97.5-percentile) contingency genes differ per strain pair and patient due to *in vivo* SSM or gene conversion from a total of over *N* = 250 (2.5-percentile) contingency genes. We finally assume an equal and independent probability for all these loci to mutate during the course of an invasive infection and that all changes are selectively neutral. The result of phase-variation in strain-pairs from IMD patients can be modelled by a random experiment in which the result of each draw (the finite number of contingency genes that differ between the throat and the corresponding blood isolate) can be classified into one of two mutually exclusive categories (whether they code for Tfp function or not) and the sampling occurs without replacement. Accordingly, out of *N* = 250 contingency genes of which *K* = 7 are involved in Tfp function *n* = 3 are randomly drawn without replacement. The probability *p* that at least one gene (*k*) involved in Tfp biosynthesis is drawn by chance, is given by the hypergeometric distribution *H*(*k*;*N*;*K*;*n*) as *p* = *H*(*k*>0;250;7;3) = 0.081.

Considering the population of IMD patients as large and practically infinite, the probability *P* that at least two (*m* ≥ 2) of the *M* = 4 blood-throat pairs differ in at least one contingency gene involved in Tfp function can therefore be calculated by a binomial distribution *B*(*m*;*M*;*p*) as P = B(*m*≥2;4;0.081) = 0.0352.

### *In vitro* passage experiments with strain WUE2121

To experimentally assess the potential impact of repeated *in vitro* cultivation on the variability of genes that were found to differ between throat and blood isolates (Table 2), we performed *in vitro* cultivation experiments on Columbia blood agar plates (bioMérieux) using the semiquantitative streak method. For these *in vitro* cultivation experiments, we used the throat isolate WUE2121 as the WUE2121/WUE2120 pair showed the highest number of differences of all four strain pairs. We assumed that each strain was first streaked out in the diagnostic laboratory on an agar plate from a positive blood culture bottle according to standard recommendations on the diagnosis of acute bacterial meningitis [53] (passage #1) and subcultivated a second time on another agar plate for the preparation of a swab to be sent to the National Reference Laboratory for Meningococci for strain typing (passage #2). After receiving the swab each strain is routinely subcultivated on an agar plate (passage #3) from which another subculture is prepared for long-time storage in a freezer (passage #4). From this stock, bacteria are taken and cultivated on an agar plate for isolation of genomic DNA and sequencing (passage #5). Consequently, we subcultivated in parallel 10 colonies from an initial culture of strain WUE2121 five times over Columbia blood agar plates, transferring either single colonies from the third streak area as is common practice in routine diagnostic laboratories, or colonies from the confluent first streak area. With the exception of the region containing the Mu-like prophage we resequenced all genes that were found to differ in any of the four strain pairs compared, i.e., in the initial strain prior subcultivation and in 10 descendant clones after subcultivation.

**Table 2:** Genomic differences in blood isolates relative to the corresponding throat isolates. For each gene and locus, the inferred phenotypes of the blood isolates at the protein level are given in parentheses and below the resulting expression or functional changes (“on” or “off”).

Process	Locus/Gene	DE8539	DE8678	DE10445	WUE2120
**Illegitimate recombination**					
	Mu-like prophage	-	-	-	ΔMu
					(nd*)
**Homologous recombination (gene conversion)**					
	*tpsS1*	-	-	T1739009A	-
				A1739015G	
				A1739018G	
				G1739138A	
				G1739144C	
				(silent)	
	*pilE*	-	-	G16384T	-
				G16419T	
				C16452T	
				G16456T	
				A16481T	
				G16483T	
				G16488T	
				T16489C	
				(alternative C-terminus)	
**Slipped-strand mispairing**					
	*pilC1*	G_10_378962G_9_	-	G_10_1833654G_9_	G_10_376831G_9_
		(truncated protein)		(truncated protein)	(truncated protein)
		on→off		on→off	on→off
	*pglI*	-	-	-	G_10_386208G_11_
					(defective glycosylation)
					on→off
	*fetA*	-	C_9_2051444C_10_	-	-
			(altered promoter)		
	WUE2121_964	-	-	-	[GCC]_3_1037013[GCC]_2_
					(ΔArg646)
					on→on
	*modA12*	-	[AGCC]_14_1473315 [AGCC]_15_	-	[AGCC]_12_1290183 [AGCC]_11_
			(silent)		(silent)
			off→off		off→off

*no data

### Cell culture assays

Detroit-562 cells (ATCC CCL-138) are epithelial cells derived from a human Caucasian pharynx carcinoma [54] and were cultured according to the supplier’s instructions in MEM Eagle Medium with Earle's BSS and L‐glutamine (Lonza, Cologne, Germany) supplemented with 10% heat inactivated fetal bovine serum (Lonza), 1% sodium pyruvate (Lonza) and 1% non‐essential amino acid solution (Lonza) (EmEm+++). EAhy926 (ATCC CRL-2922) is a human umbilical vein endothelial cell line and was cultivated in DMEM (Lonza) with L-glutamine (Lonza) and 10% heat inactivated fetal bovine serum (Lonza). Cell cultures were incubated in a humid atmosphere at 37°C with 5% CO_2_ and grown in T75 flasks (Sarstedt, Nümbrecht, Germany) to a 90%-100% confluent monolayer. Four days before infection, the cells were split and seeded into 24-well tissue culture plates (Sarstedt) in 1 ml medium. Cells were grown to a density of 4x10^5^/ml cells per well. Prior infection, bacteria were pre-cultured in the respective cell culture medium for 30 min– 45 min and adjusted to a concentration of 4x10^6^ colony forming units (CFU) per ml as determined by plating appropriate dilutions on Columbia blood agar plates (bioMérieux). All infection assays were carried out in a total volume of 1 ml cell culture medium at a multiplicity of infection (MOI) of 20, and the number of cell-adherent and -invasive bacteria was determined after 4 h of infection. The number of CFU of the infection inoculum was controlled for each infection experiment individually. After the incubation period, infected cells were washed three times with the appropriate cell culture medium. The number of cell- adherent bacteria was determined by lysis of infected cells with 1% saponin (Serva, Heidelberg, Germany) for 15 min and subsequent CFU determination. Intracellular bacteria were determined after 4 h of incubation with cell culture medium containing gentamicin (Roche) at a concentration of 400 μg/ml. For each throat-blood isolate pair we calculated the CFU ratio of the adherent bacteria of the blood isolate relative to the throat isolate (adhesion ratio). For each isolate, we further calculated the CFU ratio of the invasive bacteria relative to adherent bacteria (invasion rate). From the invasion rates, we calculated the invasion ratio as the ratio of the invasion rate of the blood isolate relative to the invasion rate of the corresponding throat isolate. All experiments were done in triplicate. For the comparison with the adhesion data from Rytkönen et al. [55] the % adherence data as given in Rytkönen et al. [55] were taken to calculate the ratio of the % adherence of blood relative to the corresponding upper respiratory tract isolates.

### Lipopolysaccharide detection by silver staining following polyacrylamide electrophoresis

Lipooligosaccharide (LPS) electrophoresis was used to compare the LPS pattern between throat and corresponding blood isolates and was carried out as described in [56]. Briefly, bacteria were grown over night on Columbia blood agar plates (bioMérieux). A 1-ml bacterial suspension in phosphate buffered saline (PBS) (1.5 mM KH_2_PO_4_, 2.7 mM KCl, 8.5 mM Na_2_HPO_4_, 137 mM NaCl, pH 7.4) at an optical density at 600 nm (OD_600_) of 0.6 was pelleted at 13,000 × *g* for 1 min, and pellets were taken up in 50 μl lysis buffer (2% SDS, 4% β-mercaptoethanol, 10% glycerol, 1 M Tris [pH 6.8], bromophenol blue). The samples were boiled for 10 min and then cooled to 60°C. Then, 10 μl of proteinase K (Sigma-Aldrich) solution (2.5 mg/ml in lysis buffer) was added and samples were incubated at 60°C for 1 h. Equal volumes of sample and 2× Tricine sample solution (4% SDS, 12% glycerol, 50 mM Tris [pH 6.8], 2% β-mercaptoethanol, 0.01% Serva Blue G [Serva]) were mixed and boiled for 5 min, and then 3 μl per lane was loaded onto a Tricine-buffered polyacrylamide gel (16.5% acrylamide, 6% bis-acrylamide). After electrophoresis, gels were stained by silver staining as described in ref. [56] and photographed using a Bio-Rad ChemiDoc MP imaging system.

### Immunoblotting

Bacteria were grown in proteose peptone medium supplemented with Polyvitex (bioMérieux, Nürtingen, Germany) (PPM+) at 37°C to an OD_600_ of 0.5–0.6 using a WPA Biowave CO8000 Cell Density Meter (Biochrom Ltd., Cambridge, UK) corresponding to the mid exponential growth phase. One ml of culture was harvested by centrifugation and the pellet was resuspended in 50 μl sample buffer (5% ß-mercaptoethanol, 2% sodium dodecylsulphate (SDS), 12.5% glycerol, 0.5 M Tris-HCL pH 6.8) (all from Sigma-Aldrich, Taufkirchen, Germany) and boiled for 10 min in a boiling water bath. All samples were boiled in sample buffer at 95°C for 5 min before electrophoresis was performed on a Mini-Protean 3 apparatus (BioRad, München, Germany). Proteins were transferred from the gel onto Protran^®^ nitrocellulose membranes (Sigma-Aldrich) and were identified with SM1 antibody (diluted 1: 4000) [57] that is specific to the epitope 49–53 found in PilE of class I meningococcal pilins, or with 4B12/C11 antibody (diluted 1: 1000) [58] which recognizes meningococcal Opa proteins. Overlay with the primary antibody was followed by horseradish peroxidase (HRP)—conjugated goat anti—mouse IgG/M antibody (H&L) (Dianova, Hamburg, Germany). The filter was finally developed by use of the Pierce^®^ ECL kit (ThermoScientific, Rockford, USA).

### Serum resistance assays

Serum resistance assays were performed as described in ref. [59] in 350 μl Veronal buffered saline (5 mM barbital, 145 mM NaCl, 2.5 mM MgCl_2_ **·** 6 H_2_O, 0.5% bovine serum albumin) at 37°C with shaking and an inoculum of about 10^5^ bacteria/ml. Prior exposure to pooled human serum bacterial strains were grown in PPM+ medium at 37°C to an OD_600_ of 0.1. Serial dilutions were plated out on Columbia blood agar plates (bioMérieux) to estimate the CFU at the start of the experiment (0 min) and after 60 min of incubation at 37°C in the presence of 10% pooled human serum. Each experiment was performed in triplicate (technical replicates). The relative recovery (*RR*) was calculated as the number of *CFU* after 60 min relative to the *CFU* at 0 min according to: *RR* (60 min) = *CFU* (60 min)/*CFU* (0 min). From the RRs of the blood and corresponding throat isolates, the selection rates (SR) were calculated according to: *SR* (60 min) = *RR*_blood isolate_ (60 min)/*RR*_throat isolate_ (60 min) [15].

### Determination of *ex vivo* survival rates in human whole blood

Human *ex vivo* whole blood assays were performed according to ref. [60] with venous blood samples from one female and four male adult volunteers (age range 25a–57a). Blood donors were selected based on (a) not having a history of vaccination against *N*. *meningitidis* serogroups A, B, C, W and Y, respectively, (b) not haven taken any antibiotics within 5 days prior sampling, and (c) not currently being carriers of meningococci. The ethics committee at the Medical Faculty of the University of Würzburg approved the study (no. 237/10, 11.01.2011) and written, informed consent was obtained from all blood donors.

Prior exposure to human venous whole blood containing 5 U/ml Heparin bacterial strains were grown in PPM+ medium at 37°C to an OD_600_ of 0.5–0.6. One milliliter of the culture was harvested by centrifugation and after washing with PBS, the bacterial pellet was resuspended in 1 ml of PBS. Ten microliter of this suspension corresponding to ~ 10^6^ CFU/ml were inoculated in 1 ml human whole blood and incubated at 37°C with shaking. Aliquots were taken out after 30 minutes and serial dilutions were plated out on Columbia blood agar plates (bioMérieux) to estimate the number of viable bacteria. For each strain and donor combination the *ex vivo* survival assays were performed in triplicate (technical replicates). From each experiment, the *RR* value was calculated from the number of *CFU* after 30 min relative to the *CFU* at 0 min according to: *RR* (30 min) = *CFU* (30 min)/*CFU* (0 min). From the RRs of the blood and corresponding throat isolates, the SRs were calculated according to: *SR* (30 min) = *RR*_blood isolate_ (30 min)/*RR*_throat isolate_ (30 min) [15].

## Results

### SSR loci in *N*. *meningitidis* are enriched for genes involved in bacteria host interaction

**Fig 1:**
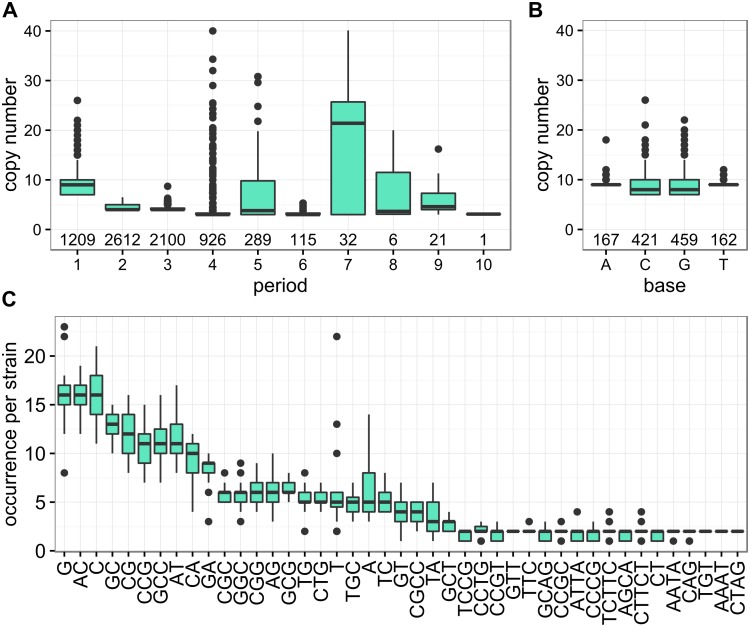
Structural properties of SSR loci in 27 meningococcal genomes. A) Characterization of SSRs by period length and copy number across all 27 assessed *N*. *meningitidis* genomes. The number of SSRs for each period length is indicated below the box-plots. B) Copy number distribution of the four different SSRs of period length one(A, C, G and T homopolymer tracts) across all 27 assessed *N*. *meningitidis* genomes. The number of SSRs for each base is indicated below the box-plots. C) Occurrence of the most frequent repeat motifs per strain, ordered by median occurrence per strain. Only motifs with a median occurrence per strain of greater than 1.5 are displayed.

Given the high genetic diversity of meningococci [5], we computationally identified SSR loci in 27 completely sequenced genomes (S3 Table) and assessed their structural properties (Fig 1 and S2A–S2C Fig).

In line with previous findings [35, 36, 61, 62], the ten most frequent SSR motifs, occurring between 10 and 20 times per genome, were homopolymeric tracts of Gs and Cs, the dinucleotides AC, GC, CG, AT, CA and GA, and the trinucleotides GCC and CCG. The majority of SSR loci contained only one or two different SSR motifs. The copy number varied widely for most repeats but showed only little variability for di- and tri-nucleotide repeats. Of note, for period lengths greater than four, the median copy number lies above the cut-off of at least three copies, indicative of selection for longer tract lengths. The median number of coding sequence (CDS) associated SSRs per genome was 273 (95%-confidence interval (CI) = [250; 297]) of which 219 (95%-CI = [206; 241]) were located within coding sequences and 49 (95%-CI = [36; 64]) within 100 bp upstream of the corresponding downstream CDS. Consequently, about 13% (95%-CI = [12%, 15%]) of all CDSs are SSR loci and could thus be targets for phase variation due to SSM in the promoter or CDS regions.

All 27 meningococcal strains further shared a set of 73 (95%-CI = [55, 96]) genes containing SSRs either within or upstream of their CDS (S2D Fig). Likewise, in all 27 meningococcal strains taken together we identified 621 (95%-CI = [458, 799]) different SSR loci that could be potential targets for phase variation via SSM. Such a large repertoire of potential phase variable genescan be explained by generation of SSRs via mutation [12] and/or by transformation and consecutive homologous recombination of SSRs into the chromosome [63]. However, we also note that this number is susceptible to artificial inflation by unrecognized orthologous genes for example due to imperfect annotation or sequencing artefacts.

**Fig 2:**
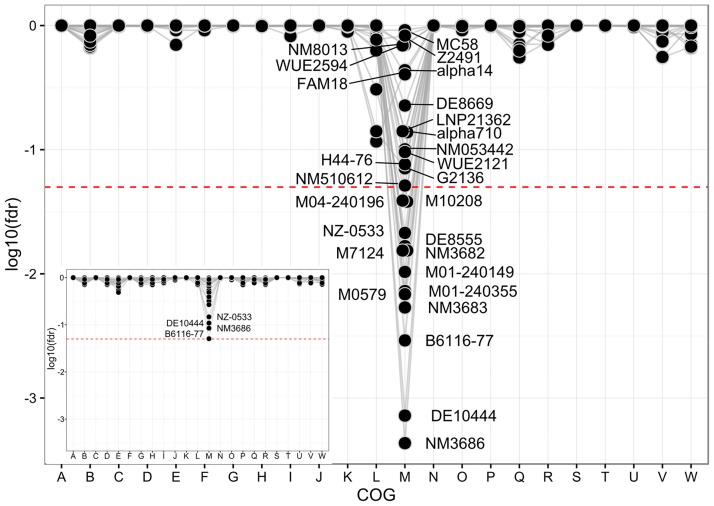
COG enrichment analyses of SSR loci in 27 meningococcal genomes. Enrichment of the different COG functional classes (B-V) among the proteins encoded by SSR loci. In the main panel, SSR loci associated with repeats having a period length of (or multiple of) three were excluded prior enrichment analysis, while in the insert the complete set of SSR loci has been analyzed. The red dashed line indicates the significance threshold (fdr < 0.05). Since genes belonging to the COG class Y (Nuclear structure) are absent from the presented dataset it has been omitted from the figures as well as all genes that do not belong to any COG functional category (often referred to as COG X). COG abbreviations: B, Chromatin structure and dynamics; C, Energy production and conversion; D, Cell cycle control, mitosis and meiosis; E, Amino acid transport and metabolism; F, Nucleotide transport and metabolism; G, Carbohydrate transport and metabolism; H, Coenzyme transport and metabolism; I, Lipid transport and metabolism; J, Translation; K, Transcription; L, Replication, recombination and repair; M, Cell wall/membrane biogenesis; N, Cell motility; O, Posttranslational modification, protein turnover, chaperones; P, Inorganic ion transport and metabolism; Q, Secondary metabolites biosynthesis, transport and catabolism; R, General function prediction only; S, Function unknown; T, Signal transduction mechanisms; U, Intracellular trafficking and secretion; V, Defense mechanisms; W, Extracellular structures.

With respect to the functions potentially affected by SSM, SSR loci could be found in almost all COG functional classes including those involved in metabolism (Fig 2). However, when excluding all SSR loci consisting of three nucleotide repeats and thus only assessing SSR loci that always result in frameshift mutations upon SSM, the remaining set of SSR loci was significantly enriched exclusively for genes involved in cell envelope biogenesis (COG M) (Fig 2). As shown in S4 Table, these included genes involved in lipooligosaccharide (*lgtA*, *lgtC*, or *lpxB*) and peptidoglycan (*murF*, *murG*) biosynthesis, respectively, as well as the genes for the immunoglobulin A protease (*iga*), the major outer membrane porin (*porA*), the minor adhesins (*opa*) and the type IV pilus glycosylation (*pglA* and *pglE*). Since these genes are all central for the interaction with the human host [3, 64, 65], this finding supports the hypothesis that phase variation due to SSM at SSR loci evolved as an adaptation for colonizing diverse hosts as required by the hypothesis of WHE [10].

### Genome sequencing of *N*. *meningitidis* throat-blood isolate pairs from IMD patients

To assess meningococcal genomic changes during IMD we sequenced throat-blood isolate pairs from four independent patients (Table 1). All throat swab and corresponding blood culture isolates were sampled within less than 24h on the day of admission. There were two male and two female patients, and the age range was 2 to 24 years. Three of the four strain-pairs belonged to the two hyperinvasive lineages (clonal complex ST-11 and ST-41/44) which cause the major share of IMD cases in industrialized countries [5]. The sample therefore has a typical composition with respect to the age and sex of IMD patients as well as the genotypes of the disease causing isolates.

**Fig 3:**
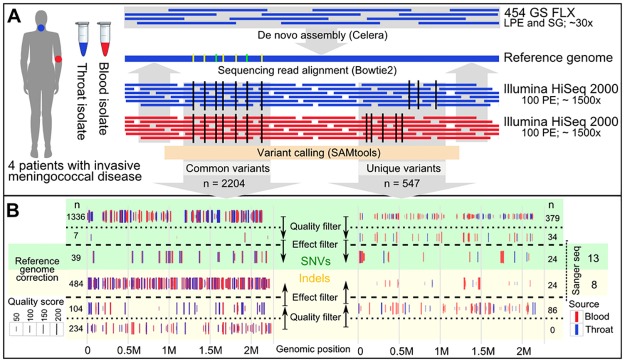
Comparative genome sequencing of meningococcal throat-blood isolate pairs from four IMD patients. (A) Reference genome assembly for the throat isolate of each patient was performed from large paired-end (LPE) libraries and whole-genome shotgun (SG) libraries produced using the NGS platform 454 Genome Sequencer FLX (GS FLX). For throat and blood isolates 100 base pair paired-end (PE) libraries were produced using the Illumina HiSeq 2000 next generation sequencing platform. (B) Common and unique variants are displayed by vertical lines according to their genomic position separated in different panels for single nucleotide variants (SNVs) and short insertions and deletions (Indels). Colors indicate the source of the variant (blue: throat; red: blood). The line height indicates the variant quality score assigned by the variant caller.

In order to assemble high-quality reference genomes, throat isolates were sequenced on the Roche 454 GS FLX platform taking advantage of the long read lengths of up to 1 kb with an average coverage of 30-fold per genome (S1 Table). For the purpose of variant analysis, all isolates were additionally sequenced on the Illumina HiSeq 2000 platform yielding an average coverage of 1500-fold per sample. The Illumina reads were mapped to the corresponding assembled throat isolate genomes and variant calling was performed on these alignments yielding in total 2751 variants before any filtering (raw variants) of which 547 were either detected in the throat or the blood isolate genomes (unique variants), and 2204 were detected in the throat as well as the blood isolate genomes (common variants) (Fig 3A). While unique variants represent potentially true differences between throat and blood isolates, common variants indicate sequencing-method specific errors in the reference genomes. The precise numbers of identified common and unique raw variants per strain, as well as the assigned variant quality scores are given in S1A Fig and Fig 3A These raw variants were filtered for quality and effect using a filtering strategy that was designed to be selective for common variants and sensitive for unique variants (see Materials and Methods) (S1B Fig and Fig 3B).

After filtering, we identified 523 common variants (39 single nucleotide variants (SNVs) and 484 insertions/deletions (indels)) and corrected the respective positions on the reference genomes accordingly (Fig 3B). The number of raw unique variants varied widely among the isolate pairs (66–190 raw variants) (S1A Fig). All unique variants that passed the quality as well as effect filter (24 SNVs and 24 indels, see Methods for details on variant filtering) were subjected to validation by Sanger sequencing by which 13 SNVs and eight indels could finally be confirmed (Fig 3B, S2 Table). This relatively low validation rate confirms our variant filtering strategy that accepted a high false positive rate in order to not falsely dismiss potential variants.

### Genotypic and inferred phenotypic differences between throat and blood isolates

Irrespective of possible mutations due to *in vitro* cultivation prior genome sequencing (discussed below), we observed genotypic differences predominantly at SSR loci in all four cases of IMD (95%-CI = [39.8%, 100%], binomial test) (Table 2 and S2 Table). On average, each strain pair differed in 7 nucleotides comprising substitutions and small indels (95%-CI = [1, 14]) and in a larger deletion of 40 kb in the case of WUE2120/WUE2121. These genomic differences can most parsimoniously be explained by 11 mutational events affecting eight different loci, resulting on average in three mutational events per strain pair (95%-CI = [1, 5]). In contrast to a previous case report of a laboratory infection with a *mutS* deficient mutator strain [18], we could not detect any mutations in mismatch repair genes in any of the four strains pairs. Considering that mutator strains with defects in the mismatch repair pathway display 10–100-fold higher phase variation rates compared to non-mutator strains [66], the number of mutations reported here is therefore in good agreement with the 30 mutations described in the aforementioned work [18]. Together, these 11 mutational events comprised eight SSM and three recombination events due to gene conversion. Five mutations, however, are silent and not expected to result in changes in the encoded protein (Table 2 and S2 Table).

Seven of the eight SSM events were located within the CDSs of either *pilC1* (three pairs), *pglI* (one pair), *modA12* (two pairs), or a phage-tail encoding gene (WUE2121_964, one pair), while one was located in the promoter region of *fetA*.

The mutations in the SSRs of *fetA* (which encodes an iron-regulated ferric enterochelin receptor, [67]) and *modA12* (coding for a phase-variable type III methyltransferase, [68]) are not expected to result in expression differences between the respective throat and blood isolates as indicated by available data [68, 69]. Changes in the SSR tract length in *pilC1*, however, lead to the generation of a truncated Tfp tip protein PilC1. The Tfp is a surface structure that promotes, amongst others, biofilm formation and adhesion to host cells and has an important role in meningococcal colonization as well as IMD [64, 65]. While PilC1 Tf pacts as an adhesin Tfp [70, 71] PglI is involved in pilin glycosylation [72] and affects Tfp the resistance to human serum complement [73] as well as the invasiveness of the respective strain [74, 75].

The three detected recombination events comprised one site specific excision of a Mu-like prophage resulting in a previously observed 40 kb deletion in the blood isolate WUE2120 [76]. Notably, computational prediction of the subcellular localization of all proteins encoded by the WUE2121 prophage identified only two outer membrane proteins. WUE2121_1250 belongs to the TonB dependent/Ligand-Gated channels, and WUE2121_1271 is a predicted D tail protein. However, both have no homologies to known meningococcal adhesins, and the same prophage was also found in the blood isolate DE8539, indicating that excision of this prophage is dispensable in IMD.

The other two recombination events were due to gene conversion, which is also stochastic and results in high-frequency antigenic variation [63]. Gene conversion occurs via intragenomic homologous recombination from silent into expression sites. Here, it affects the haemagglutinin-like adhesin TpsA1, and the major Tfp subunit protein PilE. TpsA1 is part of a two-partner secretion system and was shown to contribute to meningococcal adhesion to epithelial cells [77] and to be required for their intracellular survival [78]. The genomes of DE10444 and DE10445 both contain a *tpsS1* silent cassette with an identical sequence stretch as in the *tpsS1* expression site of the blood isolate DE10445 and different form the corresponding sequence stretch in the expression site of the throat isolate DE10444. However, gene conversion in *tpsA1* comprised only silent substitutions and the encoded protein is therefore identical in strains DE10444 and DE10445. Gene conversion of *pilE*, in turn, resulted in different PilE proteins in DE10444/DE10445 either due to horizontal transfer of a C-terminal cassette from another *Neisseria* species [79] or due to two intragenomic recombination events from two different *pilS* cassettes. The observation that the SM1 antibody only recognized PilE from DE10445 but not from DE10444 (S3 Fig) suggest that this gene conversion event is likely associated with antigenic variation also in the human host. Although we could not find any evidence for such a gene conversion event affecting the *pilE* locus in any of the other three genome pairs, the finding that *pilE* and *pilC1* are subject to phase and antigenic variation during IMD is in line with previous findings in upper respiratory tract-invasive isolate pairs from eight patients [55]. Detailed genome comparisons further failed to reveal any differences in other phase or antigenic variable genes between throat and blood isolates such as Opa or the LPS which both play a critical role in the interaction of meningococci with human host cells [80]. These genotypic findings were also supported by Opa immunoblotting as well as LPS electrophoresis which indicated that all throat and blood isolate pairs were isogenic for these particular phenotypes (S3 Fig).

Altogether, all genomic changes occurred exclusively in loci subject to SSM or gene conversion and predominantly affected genes involved in host interactions but not any housekeeping genes.

### Genetic changes due to *in vitro* cultivation of strains prior sequencing

Since all strains have been cultivated *in vitro* before sequencing, it is possible that the mutations particularly at SSR loci have, at least in part, arisen during cultivation. Assuming a standard diagnostic workflow, each strain was passaged about five times between the first isolation from a positive blood culture or throat swab in the primary diagnostic laboratory and DNA preparation for genome sequencing. In order to assess the impact of *in vitro* cultivation on sequence variability at SSR loci experimentally, we passaged strain WUE2121 five times over night on Columbia blood agar plates using the semiquantitative streak method by transferring single colonies from the third streak area onto a fresh agar plate as is common practice in routine diagnostic laboratories. We used the throat isolate WUE2121 because the WUE2121/WUE2120 pair showed the highest number of sequence differences of all four strain pairs compared (Table 2). To avoid repeated genetic bottlenecks due to single colony transfer which is known to purge any genetic diversity that might be present in the sample, we additionally transferred colonies from the confluent first streak area in a parallel experiment. With the exception of the region containing the Mu-like prophage which was already shown in an independent study to differ between WUE2121 und WUE 2120, we sequenced all genes that were found to differ in any of the four strain pairs (Table 2) in ten randomly picked colonies from the last passage. As shown in Table 3, we could not detect any sequence differences in both transfer protocols in *tpsS1*, *pilE*, *pglI* and WUE2121_964, and only one clone with a variant *modA12* in the single colony transfer experiment. However, we found a high percentage of colonies with a mutated *fetA* due to serial cultivation in both transfer protocols. In addition, while there were no differences in the *pilC1* sequences between the original and the descendant clones after repeated single colony transfer, the majority of descendant colonies differed from the original clone following transfer from the confluent first streak area. In fact, only the effect of the mode of transfer on the observed genetic variation was significant (p = 0.014, logistic regression analysis). The average probability of a variant gene sequence due to *in vitro* cultivation was 0.07 (95%-CI = [0.00, 0.36]) per locus after single colony transfer and 0.20 (95%-CI = [0.00, 0.77]) per locus after transfer from the confluent first streak area, respectively.

**Table 3:** Gene sequence differences in ten colonies of strain WUE2121 after five *in vitro* passages using the semiquantitative streak method compared to the initial clone.

	Single-colony transfer from third streak area		Transfer from the confluent third streak area	
Locus/Gene	Fraction of mutated colonies	95%-CI	Fraction of mutated colonies	95%-CI
**Homologous recombination**				
*tpsS1*	0.0	[0.00, 0.31]	0.0	[0.00, 0.31]
*pilE*	0.0	[0.00, 0.31]	0.0	[0.00, 0.31]
**Slipped-strand mispairing**				
*pilC1*	0.0	[0.00, 0.31]	0.6	[0.26, 0.88]
*pglI*	0.0	[0.00, 0.31]	0.0	[0.00, 0.34]
*fetA*	0.5	[0.22, 0.86]	0.8	[0.40, 0.97]
WUE2121_964	0.0	[0.00, 0.31]	0.0	[0.00, 0.31]
*modA12*	0.1	[0.00, 0.45]	0.0	[0.00, 0.31]

To further assess whether the high sequence variability of *fetA* and *pilC1* was already present in the inoculum prior to serial transfer both genes were also sequenced in colonies cultivated directly from the glycerol stock. As depicted in S5 Table the coefficient of variation for the poly-C tract length of *fetA* was 10% among colonies from the inoculum which was comparable to the 11% obtained for the coefficient of variation of *fetA* poly-C tract lengths after single-colony transfer. The corresponding coefficient of variation for the poly-G tract length of *pilC1* was 3% among colonies in the inoculum and 0% after single-colony transfer. Therefore, at least in the case of *fetA* a high poly-C tract length variability seems to be present already in the inoculum.

In consequence, when excluding *fetA* as well as *pilC1* because of their high *in vitro* variability, strain pairs differed *in vivo* either due to gene conversion or SSM on average in one gene (95%-CI = [1, 3]) in about half of the patients (95%-CI = [7%, 93%]).

### Mutations predominantly affect the type IV pilus

As noted above, mutations in *tpsA1*, *fetA* and *modA12* were all silent in contrast to the mutations in *pilE*, *pilC1* and *pglI*, which are all involved in Tfp biogenesis, and we could not detect any changes in other invasion-associated genes. Functional genomics analysis has shown that 23 genes are involved in Tfp biogenesis, of which seven are subject to phase variation either via SSM or gene conversion, respectively [36, 41]. Although the fraction of such variable genes is somewhat higher among the Tfp biogenesis genes compared to genes coding for other functions (23% vs. 14%, OR = 1.60, 95%-CI = [0.53, 4.07]), the difference is yet not significant (p = 0.284, Fisher’s exact test). The location, sequence or length of SSRs were also not significantly different between *pilC1* and *pglI* genes and contingency genes like, e.g., *lgtA*, *iga* or *porA* that did not differ between strain pairs (Table 2 and S4 Table). To estimate an upper limit for the probability that the number of mutated Tfp associated genes (*k*) during IMD exceeds zero just by chance, we therefore assumed that mutations only occur in up to 3 (97.5-percentile, see above) of the over 250 different contingency loci (2.5-percentile, see above) and with an equal and independent probability. If all these changes were selectively neutral, the corresponding probability would be *p*(*k* > 0) = 0.081 (hypergeometric distribution), and the probability that the Tfp was affected in at least two of the four strains pairs (*m*) just by chance consequently would be *P*(*m* ≥ 2) = 0.035 (binomial distribution). Consequently, on a significance level of 5% mutations at contingency genes involved in Tfp biogenesis occur at a higher frequency among IMD isolate pairs than expected by chance. We therefore tested whether throat and blood isolates also differed in their within-host fitness as proposed by the theory of direct selection during WHE [9, 10].

### Differences in serum resistance between strain pairs are dependent on the strain but not their source of isolation

**Fig 4:**
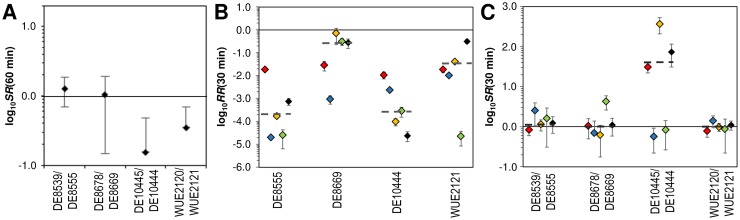
Comparison of the *ex vivo* fitness of meningococcal strains. (A) Comparison of the *ex vivo* selection rates (*SR*) of blood relative to the corresponding throat isolates after 60 min incubation in 10% pooled human serum. Mean and standard deviation are depicted, and each experiment was carried out in triplicate. (B) Relative recovery (*RR*) of throat isolates in human blood calculated from the colony forming units (*CFU*) at 0 min and 30 min according to *RR* (30 min) = *CFU* (30 min)/*CFU* (0 min) (13). (C) Comparison of *ex vivo* selection rates (*SR*) of blood relative to the corresponding throat isolates in human blood calculated as *SR* (30 min) = *RR*_blood isolate_ (30 min)/*RR*_throat isolate_ (30 min) (13). For each strain (pair) and donor, the scatter plot gives the mean and standard deviation of three independent experiments. Each donor is color-coded. For each strain (pair), dashed lines indicate the median RR and SR values, respectively, over all five donors.

Since Tfp antigenicity and/or Tfp glycosylation were shown to be important in serum resistance [73] we assessed *ex vivo* resistance to 10% pooled human serum in standard serum resistance assays. As depicted in Fig 4A only the two blood isolates with either modified Tfp antigenicity (DE10445) or with predicted differences in the Tfp glyosylation status (WUE2121) compared to the corresponding throat isolates had also a slightly decreased serum resistance, although these differences did not reach statistical significance (two-sided t tests with pooled standard deviation from three replicate experiments). When considering each strain as a biological replicate of an either throat or blood derived isolate and thus ignoring genetic differences among throat isolates and among blood isolates, respectively, there was no significant difference in serum resistance between throat and blood isolates (Wilcoxon signed rank test, p > 0.05).

### The fitness of throat and blood isolates does not differ in a human *ex vivo* blood assay

Under the assumption of direct selection during WHE, blood isolates should be better adapted to survive in human blood than their colonizing ancestors [9, 10]. Since *N*. *meningitidis* is a strictly human adapted species, we used a human *ex vivo* whole-blood model to test for fitness differences among the different isolates. Although none of the throat isolates was able to grow in blood from five healthy donors (log_10_ *RR*(30 min) < 0 in Fig 4B), there was statistically significant variation in the recovery rates with respect to the strains (Kruskal-Wallis test, p = 0.047) but not the donors (p = 0.539). The recovery rates of blood isolates were slightly higher than the ones of the throat isolates, resulting in selection rates slightly larger than 1 (log_10_ *SR*(30min) ≥ 0 in Fig 4C). However, these differences were not statistically significant (p > 0.05, two-sided one-sample t-test with Bonferroni multiple testing correction). Again, the variation of the selection rates was greater over the four strain-pairs (Pearson's chi-squared test for count data, χ² = 2.3246, p = 0.51) than over the five blood donors (χ² = 1.5833, p = 0.82), but did not reach statistical significance in either case (Kruskal-Wallis test). Finally, the median *ex vivo* selection rates for each strain pair over all five donors was not significantly greater than one (p > 0.10, two-sided one-sample Wilcoxon signed rank test with Bonferroni multiple testing correction). As all strains were isolated from IMD patients, we conclude that host genetic factors as well as the immune status of the host play a predominant role particularly in colonization and that once a meningococcal strain has successfully colonized a host and entered the bloodstream, host factors might be less important than bacterial factors for the development of IMD. In addition, in line with the fact that we could not observe any mutations in metabolic genes in the blood isolates our data reject the hypothesis that WHE selects for strains with a higher fitness in human blood (95%-CI = [0.0%, 60.2%], binomial test).

### Throat and blood isolates differ slightly in the interaction with human cells

**Fig 5:**
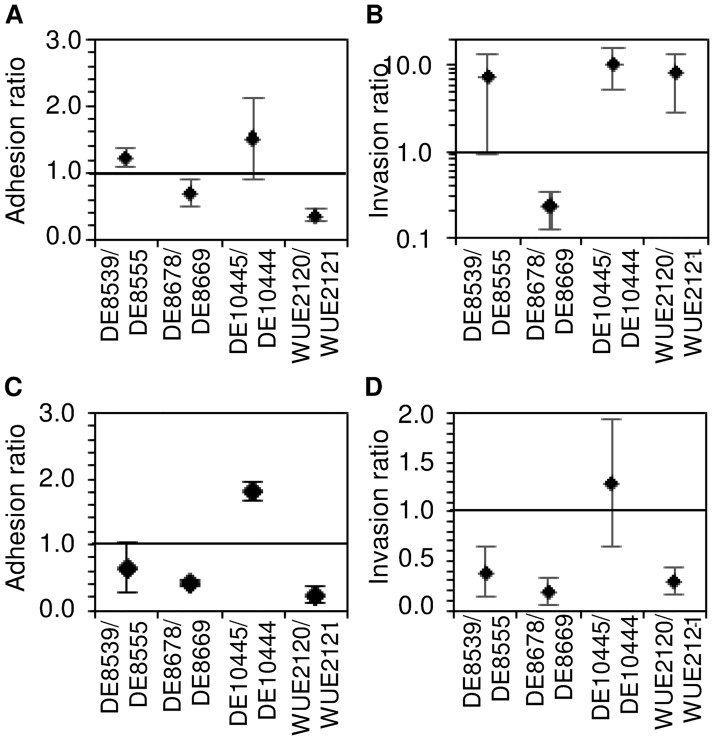
Comparison of the interaction of meningococci with human cells. Panels (A) and (B) compare the interaction of meningococcal throat and blood isolates with Detroit-562 epithelial cells derived from a human pharynx carcinoma. Likewise, panels (C) and (D) compare their adhesion to and invasion of human EAhy926 umbilical vein endothelial cells. (A) and (C) Scatter plots of the adhesion ratios for each strain pair given as the CFU ratio of the adherent bacteria of the blood isolate relative to the respective throat isolate. Values greater (smaller) than one indicate that the blood isolates adhere better (less) to human cells than the corresponding throat isolate. (B) and (D) Scatter plots of the invasion ratios for each strain pair given as the ratio of the invasion rate of the blood isolate relative to the invasion rate of the corresponding throat isolate. Values greater (smaller) than one indicate that the blood isolates are more invasive than the corresponding throat isolate. For each strain pair, the mean and standard deviation of three independent experiments are given.

Since Tfp (glycosylation) was also shown to be important for invasion of host cells [74, 75] we finally assessed the interaction of the bacterial isolates with human epithelial and endothelial cell lines for differences in adhesion and/or invasion properties *in vitro*. As can be seen in Fig 5, the adhesion ratio to human Detroit-562 nasopharyngeal epithelial cells calculated as ratio of adherent blood isolate CFU relative to the corresponding throat isolate CFU was slightly larger than one for the strain pair DE10445/DE10444 and significantly smaller than one only for the adhesion of strain pair WUE2120/WUE2121 (p < 0.05, two-sided one-sample t-test with Bonferroni multiple testing correction) Of note, the latter strain pair is predicted to differ in Tfp glycosylation based on the genomic data (Table 2). The average adhesion ratios over all four strain pairs was 0.95 ± 0.26 (SDM), indicating that there were no significant differences yet in the adhesion of throat and blood isolates to human nasopharyngeal epithelial cells *in vitro*. The fact that this number was very similar to the corresponding number of 1.02 ± 0.45 derived from data given in ref. [55] further suggests that the experimentally determined adhesion rates are robust against differences in the experimental conditions and population samples.

Similar to the adhesion assays with epithelial cell lines the two strain pairs with the largest genetic differences including predicted differences in Tfp glycosylation and *pilE* sequence, DE10445/DE10444 and WUE2120/WUE2021, respectively, also showed the largest differences in adhesion ratios to the human endothelial cell line EAhy926 among the four strain pairs (Fig 5). However, none of the four adhesion ratios was significantly different from one (p > 0.05, two-sided one-sample t-test with Bonferroni multiple testing correction). The average adhesion ratio over all four strains pairs was 0.78 ± 0.35 (SDM), indicating that blood isolates adhere slightly but not significantly less to endothelial cells than the corresponding throat isolates (p > 0.10, one-sample Wilcoxon test).

Furthermore, pairwise comparisons of the invasion ratios as defined for each strain by the ratio of the invasion rate of the blood isolate relative to the invasion rate of the corresponding throat isolate (for detailed information see Materials and Methods section in the Supporting Information) showed that with the exception of DE8678 all blood isolates were slightly but not significantly more invasive than the corresponding throat isolates in human epithelial cells (average invasion ratio = 6.66 ± 2.26) (p = 0.25, Wilcoxon test), and overall less invasive in human endothelial cells (average invasion ratio = 0.54 ± 0.25) (p = 0.25).

Given the overall very low invasion rates of less than 0.05% of the adherent bacteria as well as the very small difference between the throat and blood isolates, a higher number of such throat-blood strain pairs (n > 10) will be required to allow for statistically more robust analyses of such small phenotypic differences.

## Discussion

Several studies have already used comparative genome sequencing of longitudinal paired strain samples to provide evidence for adaptive within-host evolution mostly in pathogens that cause infections lasting days to months such as *Staphylococus aureus* or *Escherichia coli* [81, 82] (reviewed in [83]). IMD, however, is a prime example for a (per)acute bacterial infection, and patients suspected of suffering from IMD require immediate treatment usually with a third generation cephalosporin which aims at killing all live bacteria after the first dose [53]. It is thus not possible in practice to obtain longitudinal paired samples, and all throat—blood isolate pairs investigated in this study were sampled within 24 h after hospitalization from each IMD patient. The ensuing lack of multiple time-stamped data sets allows in principle for different explanations of the observed genetic differences between the throat and the corresponding blood isolates. Accordingly, the genetic variation observed in the paired strain samples might (i) have occurred during *in vitro* cultivation subsequent to strain sampling, (ii) have already existed when the individual was exposed to or colonized by a second, invasive strain, or (iii) result from WHE either during colonization or upon host invasion.

With respect to the effect of *in vitro* cultivation, mutations possibly occurring during cultivation as well as the picking of colonies from agar plates which is common practice in diagnostic laboratories can both affect the amount of genetic changes detected by genome sequencing. Based on the experimentally determined probabilities that the genetic differences observed were due to *in vitro* cultivation (Table 3), the probability that all three mutational events observed on average per strain pair were solely due to *in vitro* cultivation is less than 1%. In line with previous observations [84, 85], phase variation was particularly frequent in *fetA* and *pilC1* during *in vitro* cultivation (Table 3). However, as mentioned above, the number of mutations observed in this study is in good agreement with what has been reported from a case of a laboratory infection [18] in which genomic sequencing was directly performed on frozen stocks isolated from the bloodstream without further subculture, and also the expression status of the hemoglobin-binding proteins HmbR and Hpu were found to be stable during meningococcal isolation from patient specimens [86]. Likewise, the genetic changes in *pilC* and *pilE* as described in Table 2 have also been confirmed in a similar study assessing genetic changes in surface protein encoding genes during IMD [55]. Therefore, the comparison with published data suggests that the sequencing results are robust with respect to laboratory handling of the strains. To exclude all secondary effects of cultivation on meningococcal genetic diversity, NGS technologies will have to be improved to allow using throat swabs and blood samples directly for sequencing without any need for prior strain cultivation, including also conventional blood culture approaches routinely used for the microbiological diagnosis of IMD.

With respect to co-infection with two genetically distinct meningococcal strains, the presence of more than one clone as identified by multilocus sequence typing was detected in only about 5.5% (95%-CI = [0.3%, 10.3%]) of healthy carriers in different carriage studies (reviewed in ref. [87]). Therefore, colonization of the throat by multiple meningococcal clones is a rare event and in most cases the meningococcal flora is dominated by a single clone [87]. In particular, the prevalence of ST-11 clones among carriage isolates in Europe was found to be less than 5% and with only a short duration of carriage [87]. In addition, all four strain pairs including the two ST-11 pairs were from sporadic cases and not sampled during an outbreak, and under the assumption of random population mixing and a meningococcal carriage rate of about 10% the probability of a co-infection with another strain from, e.g., clonal complex ST-11, would be ≤ 0.5% and thus unlikely. Finally, based on the nucleotide differences given in Table 2 the pairwise genetic distances between the two ST-11 throat-blood isolate pairs WUE2121/WUE2120 and DE8555/DE8539 was around 2 x 10^−6^ which is a hundred times smaller than the average pairwise distances between genetically highly related ST-11 strains from a clonal outbreak (3 x 10^−4^, Hajj cluster, ref. [88]). In conclusion, the hypothesis that the genetic variation observed in the paired strain samples is due to a co-infection with two meningococcal strains seems rather unlikely.

With respect to the hypothesis that the genetic variation observed in the paired strain samples results from WHE, Bayliss and co-workers showed that mutants at SSR loci are indeed randomly generated in the nasopharynx during colonization [69]. By analyzing mononucleotide repeat tracts at meningococcal SSR loci during carriage they further estimated that the switching rate at contingency loci with mononucleotide repeat tracts is about 0.06 mutations/gene/month of carriage [69]. Given that there are on average 45 CDS associated mononucleotide SSRs per meningococcal genome (95%-CI = [33, 55]) (Fig 1 and S3 Table), the expected number of mutations due to SSM at such SSR loci is about 3 (95%-CI = [2, 3]) per month of carriage which is slightly more than the observed number of about 1 mutation per strain pair at such SSR loci (95%-CI = [1, 2]) (Wilcoxon test, p < 0.001) (Table 2). Although the time span between meningococcal acquisition and the onset of IMD is not known for sporadic cases, over 70% of subsequent IMD cases occur within 2 weeks of the index case in an outbreak situation [89]. Together, these data therefore suggest that SSM at SSR loci would be able to generate the genetic diversity observed in the throat-blood isolate pairs during nasopharyngeal carriage within an incubation time span that is typical for IMD, and that the genomic differences in throat and blood isolates from patients with IMD therefore most likely result from WHE [8–10]. Whether the mutational pattern is unique for IMD progression and absent in asymptomatic long-term carriage will await genome sequencing of longitudinal paired strain samples from healthy meningococcal carriers.

The low number of mutations separating throat from blood isolates in *N*. *meningitidis* is also in good agreement with similar findings in other bacterial pathogens [83]. For example, the analysis of the evolutionary dynamics during progression from long-term carriage to bloodstream infection demonstrated that only eight mutations separate disease-causing from asymptomatically carried *S*. *aureus* strains [82]. The observed association between mutations in Tfp contingency genes and IMD could result from increased antigen and phase-variation frequencies, respectively, of Tfp genes compared to other phase-variable genes due to the long repeat tract length of *pilC1* and *pglI* (Table 2). However, there are a number of genes that have homopolymeric tract lengths comparable to the tract length of *pilC1* and *pglI* like *kdtA*, *lgtA* or *opc* (S4 Table) which did not differ between throat and corresponding blood isolates. Alternatively, genetic changes affecting Tfp biosynthesis might be subject to positive selection during IMD. The cell culture experiments suggest that three of the four blood isolates did show an improvement in invasiveness compared to the throat isolate (Fig 5). This may reflect that there is increased invasiveness across the epithelial barrier to result in systemic disease, but once in this environment all strains were apparently similar in their ability to interact with endothelial cells. However, the two blood isolates DE10445 and WUE2120 had a lower resistance against human serum, and there was also no difference in survival in human blood *ex vivo* between throat and blood isolates in general (Fig 4). The fact that we could not find direct evidence for adaptive selection for increased within-host fitness is yet in line with similar findings in other commensal pathogens such as, e.g., in urinary tract infections caused by *E*. *coli* after long-term colonization [81].

With respect to the potential phenotypic effect of these mutations on meningococcal virulence, i.e. host damage, it has recently been demonstrated that the interaction of Tfp with human blood vessel endothelial cells [90, 91] as well as Tfp posttranslational modification [92, 93] both play a critical role in the pathogenesis of IMD in vivo [64]. It therefore seems possible that mutations particularly in Tfp genes might be associated with host damage not detectable by established *in vitro* and *ex vivo* virulence assays, respectively, and that virulence in meningococci consequently occurs due to accidental evolution without affecting within-host fitness [8, 14]. Since the Tfp is essential also for host colonization [65], mutations in Tfp genes generated in the nasopharynx during colonization [10, 13] might be pre-adaptive for virulent exploitation of the host [14]. The experimental test of these hypotheses might be possible with appropriate transgenic mouse models recently described [90, 91].

The *in-silico* whole-genome comparisons of 27 meningococcal strains analyzed in this work (Fig 2) further support the hypothesis that phase variation due to SSM at SSR loci evolved as an adaptation for colonization of diverse hosts [10, 13]. Mechanistically, SSM couples phase variation with replication, and strains with the ability to grow to higher densities during niche colonization would consequently have a higher probability of randomly generating mutants with an accidentally higher propensity to cause host damage. In line with this hypothesis, we recently reported that hyperinvasive lineages have significantly higher *in vitro* growth capacities than carriage lineages [94]. Phase variation due to SSM at SSR loci would therefore also provide a mechanistic explanation of how growth differences among meningococcal lineages are associated with higher virulence [95].

Of note, phase-variable gene expression has so far not been shown experimentally for all of the repeat types considered as SSR loci in this computational comparison (e.g. [61, 96]), nor do all repeat types for which phase-variable gene expression has been demonstrated display the same mutation rates (e.g. [69, 96]). Therefore, the number and types of possible SSR loci provide just an estimate for the amount of phenotypic variability that might be generated via SSM, which further depends on the choices of parameter settings that have inevitably to be made in such computational analyses. Notwithstanding these limitations, the number of SSR loci as well as the functional spectrum of the affected genes reported in this analysis is in good agreement with the results of a recent study by Siena et al. [96] which performed a similar population-scale comparative genomic analysis for SSR loci. The authors identified 277 potentially phase-variable genes, predominantly coding for cell surface components as well as for a broad spectrum of metabolic functions including DNA metabolism. Further studies are clearly required to better understand the adaptive potential of SSM during meningococcal carriage and disease. Together, our data do not provide experimental evidence for within-host selection of invasive strains as a common mechanism for IMD. They rather suggest a predominantly neutral sequence evolution of meningococci during IMD and without direct selection for increased virulence within the host.

We conclude that, although the number of strains analyzed in this work is already higher than in all recent studies addressing WHE in patients with acute invasive bacterial infections [17, 18], a yet higher number of strains from IMD patients will be required to not only allow for statistically more robust conclusions but also for the detection of rare mutations as well as small phenotypic differences between throat and blood isolates. Given the low incidence of IMD in industrialized countries with < 1/100.000/year [3] and the low frequency at which paired samples are routinely taken in cases of suspected IMD, the sampling of strain pairs from a large number of patients in a multi-center study would be required to properly address these issues.

## Supporting Information

S1 Fig Variant quality score distribution for common- and unique variants. A) Boxplots representing the distribution of the raw-variant quality scores as assigned during variant calling through samtools-vcftools for the eight assessed *N*. *meningitidis* isolates (red: blood isolate, blue throat isolate). Common- and unique variants are displayed in separate panels. Numbers below the boxplots represent the number of raw-variants identified in the respective isolate and category. B) Loess-smoothed occurrence distribution of quality scores for common and unique variants across all assessed samples. The confidence interval to a confidence level of 0.95 is indicated.
(TIF)

S2 Fig Computational analysis of SSR loci in *N*. *meningitidis*. A) Number of identified CDS associated SSRs per strain (total) and the number of CDS associated SSRs with a period length of three or a multiple of three (mult3). B) Occurrence of different numbers of SSRs (1 to 4) per CDS per strain. C) Number of CDS associated SSRs per strain that are located within the coding sequence (intra) or the promoter region (prom). D) Number of common (cSSR) and different (dSSR) SSR associated CDS among all possible combinations of the indicated number of assessed genomes (abbreviated by the letter g), drawn from a pool of 27 different *N*. *meningitidis* genomes. The vertical lines represent the maximum and minimum for each number of assessed genomes. cSSRs are those loci, that are found in all assessed genomes while dSSRs represent the entirety of all SSR loci found in any of the assessed genomes. Confidence intervals to a confidence level of 0.95 are indicated by yellow and green ribbons. The functions model the median number of cSSR and dSSR in dependence of the number of assessed genomes (g).
(TIF)

S3 Fig Phenotypic comparisons. Upper panel: Tricine gel electrophoresis of partially purified LPS. Middle panel: Expression of the Tfp major pilin protein PilE as detected with the antibody SM1 specific for class I meningococcal pilins. Lower panel: Expression of the Opa outer membrane protein with antibody 4B12/C11. Strain MC58 from the clonal complex ST-32 and expressing a class I pilus was used as positive control. Strain pairs DE8555/DE8539 and WUE2121/WUE2120, which belong to the clonal complex ST-11 and consequently express class II pili, have been used as internal negative controls for the expression of class I pilins. M: ColorPlus Prestained Protein Ladder, Broad Range (10–230 kDa) (New England Biolabs, Frankfurt/Main, Germany).
(TIF)

S1 Table 454 GS FLX sequencing of the throat isolates, and Illumina HiSeq 2000 sequencing of throat and blood isolates. (DOCX)

S2 Table Variant validation by Sanger sequencing. (DOCX)

S3 Table Strains and genome sequences used for *in silico* genome comparisons. (DOCX)

S4 Table SSR loci encoding proteins of the COG functional category M (cell wall/membrane/envelope biogenesis). (DOCX)

S5 Table Repeat tract length variation of *fetA* and *pilC1* during five *in vitro* passages of strain WUE2121. (DOCX)
