# Supplementary figures and images for: Comparative Genome Sequencing Reveals Within-Host Genetic Changes in Neisseria meningitidis during Invasive Disease
Source: PLoS One. 2017 Jan 12;12(1):e0169892. doi: 10.1371/journal.pone.0169892 (PMC5231331; doi:10.1371/journal.pone.0169892)

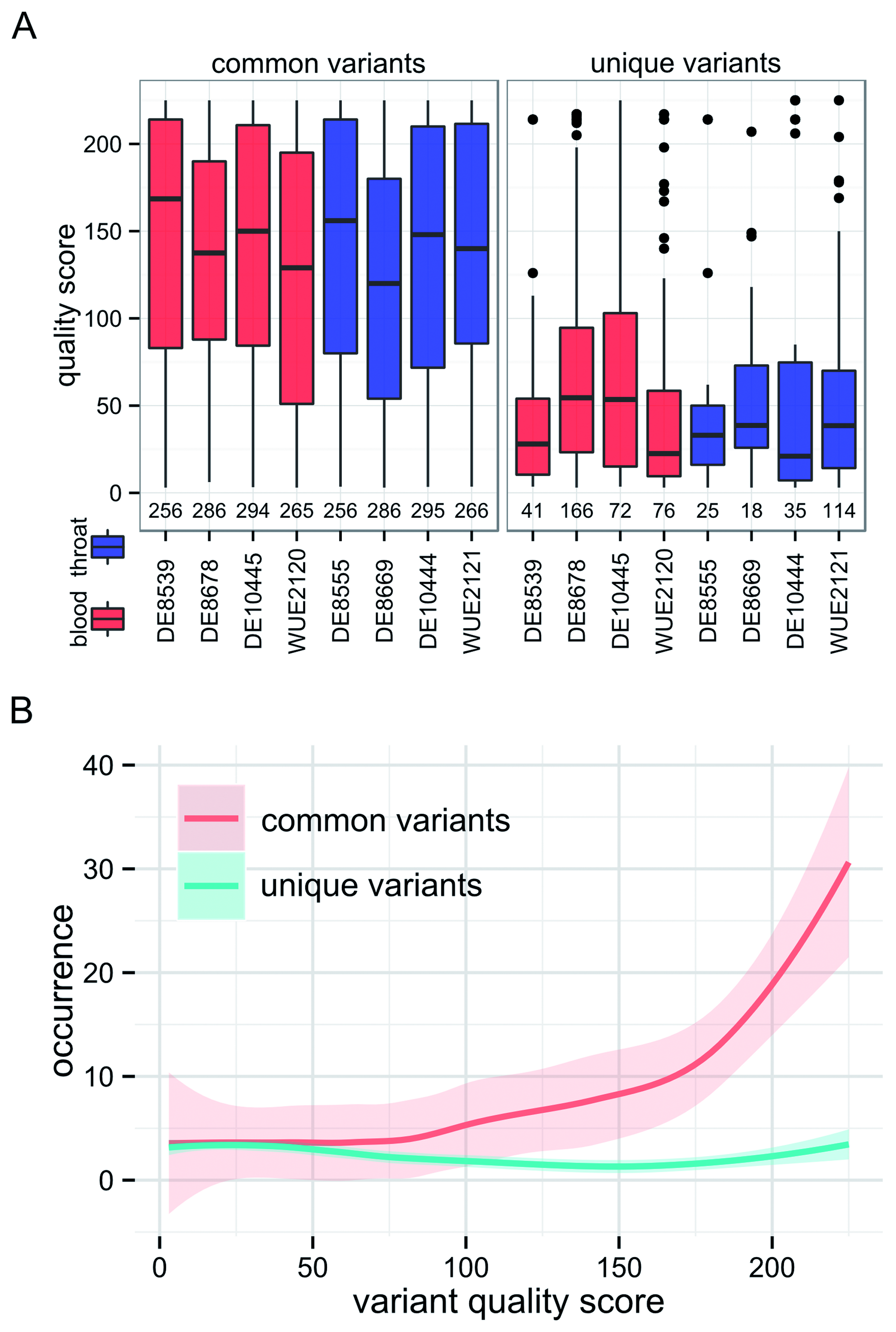

Supplement: S1 Fig — A) Boxplots representing the distribution of the raw-variant quality scores as assigned during variant calling through samtools-vcftools for the eight assessed N. meningitidis isolates (red: blood isolate, blue throat isolate). Common- and unique variants are displayed in separate panels. Numbers below the boxplots represent the number of raw-variants identified in the respective isolate and category. B) Loess-smoothed occurrence distribution of quality scores for common and unique variants across all assessed samples. The confidence interval to a confidence level of 0.95 is indicated. (TIF) [file pone.0169892.s001.tif]

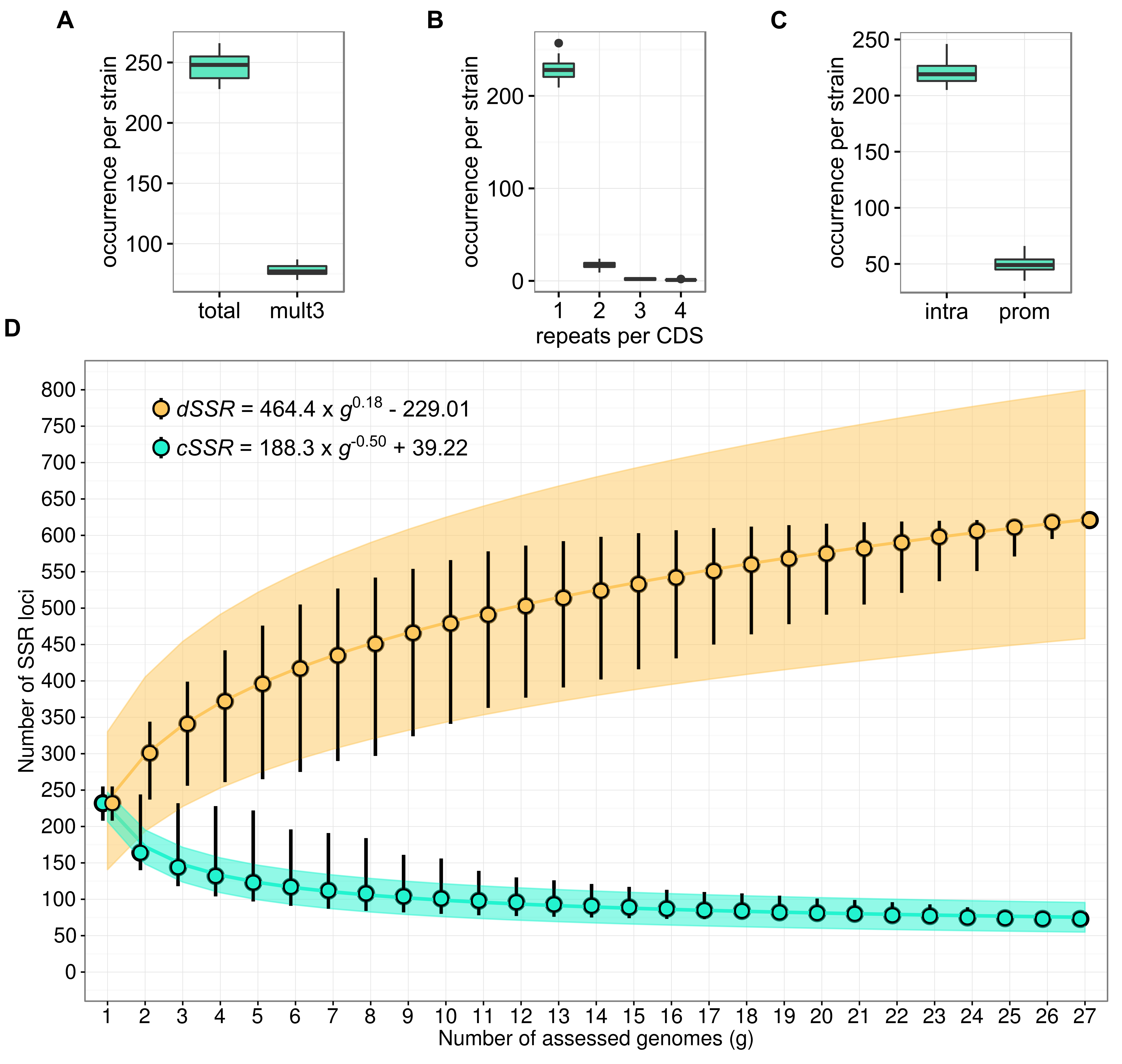

Supplement: S2 Fig — A) Number of identified CDS associated SSRs per strain (total) and the number of CDS associated SSRs with a period length of three or a multiple of three (mult3). B) Occurrence of different numbers of SSRs (1 to 4) per CDS per strain. C) Number of CDS associated SSRs per strain that are located within the coding sequence (intra) or the promoter region (prom). D) Number of common (cSSR) and different (dSSR) SSR associated CDS among all possible combinations of the indicated number of assessed genomes (abbreviated by the letter g), drawn from a pool of 27 different N. meningitidis genomes. The vertical lines represent the maximum and minimum for each number of assessed genomes. cSSRs are those loci, that are found in all assessed genomes while dSSRs represent the entirety of all SSR loci found in any of the assessed genomes. Confidence intervals to a confidence level of 0.95 are indicated by yellow and green ribbons. The functions model the median number of cSSR and dSSR in dependence of the number of assessed genomes (g). (TIF) [file pone.0169892.s002.tif]

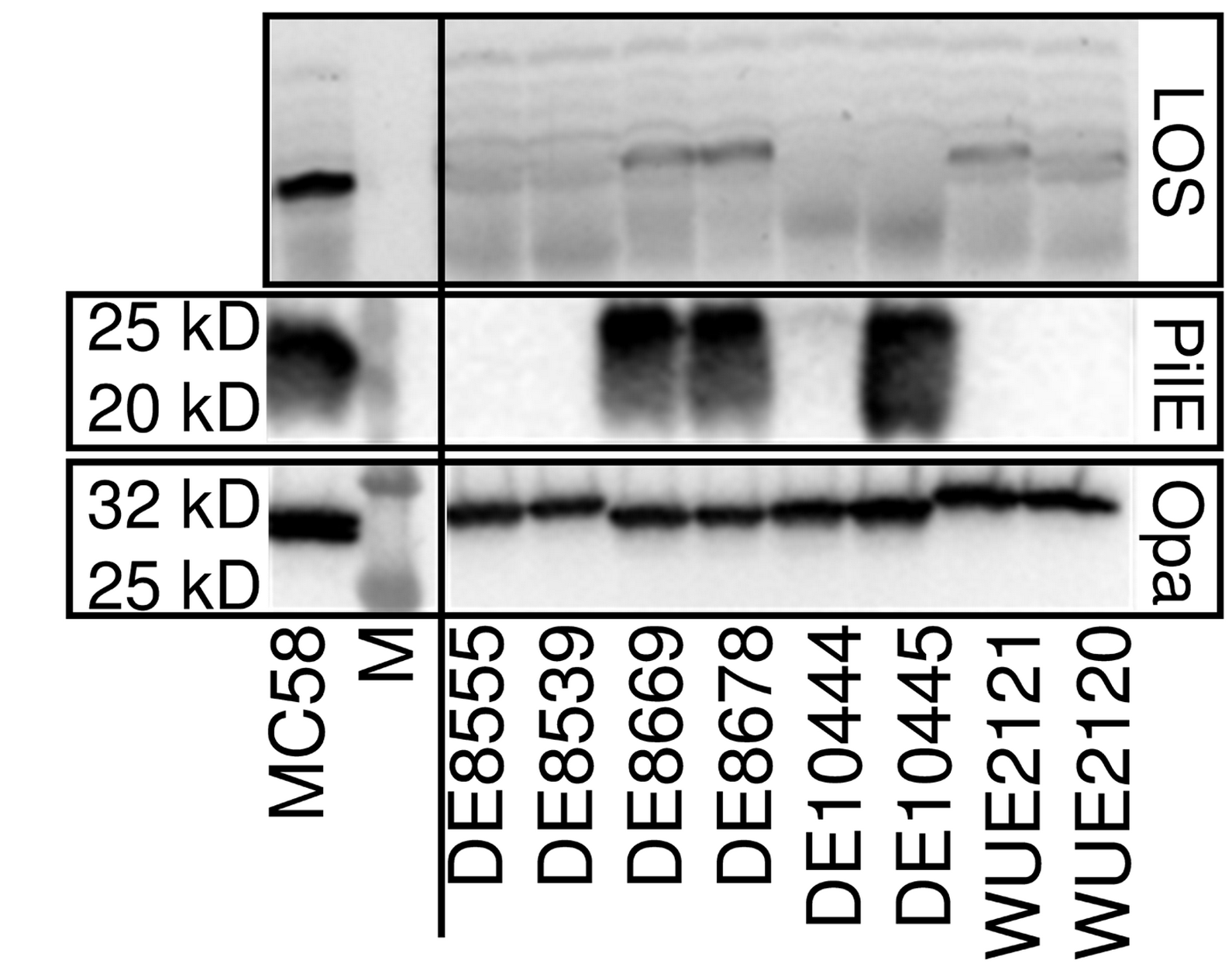

Supplement: S3 Fig — Upper panel: Tricine gel electrophoresis of partially purified LPS. Middle panel: Expression of the Tfp major pilin protein PilE as detected with the antibody SM1 specific for class I meningococcal pilins. Lower panel: Expression of the Opa outer membrane protein with antibody 4B12/C11. Strain MC58 from the clonal complex ST-32 and expressing a class I pilus was used as positive control. Strain pairs DE8555/DE8539 and WUE2121/WUE2120, which belong to the clonal complex ST-11 and consequently express class II pili, have been used as internal negative controls for the expression of class I pilins. M: ColorPlus Prestained Protein Ladder, Broad Range (10–230 kDa) (New England Biolabs, Frankfurt/Main, Germany). (TIF) [file pone.0169892.s003.tif]
