# Supplementary material for: Comparative Genome Sequencing Reveals Within-Host Genetic Changes in Neisseria meningitidis during Invasive Disease
Source: PLoS One. 2017 Jan 12;12(1):e0169892. doi: 10.1371/journal.pone.0169892 (PMC5231331; doi:10.1371/journal.pone.0169892)
Supplement: S1 Table — (DOCX) [file pone.0169892.s004.docx]

**S1 Table.** **454 GS FLX sequencing of the throat isolates, and Illumina HiSeq 2000 sequencing of throat and blood isolates.**

| **Isolate** | **Sequencing method** | **High quality read pairs** | **% Reads available for assembly (>64 bp)^1^** | **Mean read length (bp)** | **Mapping rate (%)^2^** |
| --- | --- | --- | --- | --- | --- |
| DE10444 | 454 SG**^3^** | 28471 | 100 | 401 | NA**^4^** |
|  | 454 PE**^5^** | 254930 | 74 | 309 | NA |
|  | Illumina PE | 14400129 | NA | 100 | 98.50 |
| WUE2121 | 454 SG | 24787 | 100 | 406 | NA |
|  | 454 PE | 263329 | 89 | 326 | NA |
|  | Illumina PE | 26428989 | NA | 100 | 99.00 |
| DE8555 | 454 SG | 43392 | 100 | 397 | NA |
|  | 454 PE | 262787 | 92 | 321 | NA |
|  | Illumina PE | 17739152 | NA | 100 | 98.73 |
| DE8669 | 454 SG | 21136 | 100 | 404 | NA |
|  | 454 PE | 252630 | 90 | 314 | NA |
|  | Illumina PE | 11637127 | NA | 100 | 98.55 |
| DE10445 | Illumina PE | 15594100 | NA | 100 | 98.29 |
| WUE2120 | Illumina PE | 18228983 | NA | 100 | 97.35 |
| DE8539 | Illumina PE | 16239659 | NA | 100 | 98.69 |
| DE8678 | Illumina PE | 19178705 | NA | 100 | 95.06 |

^1^ Only relevant for 454 data (used for the reference genome assembly).

^2^ Only relevant for Illumina paired-end sequencing data (mapped to the reference genomes).

^3^ SG: shotgun sequencing

^4^ NA: not applicable

^5^ PE: paired-end sequencing
