# Supplementary material for: Comparative Genome Sequencing Reveals Within-Host Genetic Changes in Neisseria meningitidis during Invasive Disease
Source: PLoS One. 2017 Jan 12;12(1):e0169892. doi: 10.1371/journal.pone.0169892 (PMC5231331; doi:10.1371/journal.pone.0169892)
Supplement: S2 Table — (DOCX) [file pone.0169892.s005.docx]

| **PV state** | | **Mutation** | **Gene** | **Forward primer**  **Reverse primer** | **Product length** | **Order** | **Sanger-sequencing alignment*** |
| --- | --- | --- | --- | --- | --- | --- | --- |
| **Throat isolate** | **Blood isolate** |  |  |  |  |  |  |
| On | Off | G_10_378962G_9_ | *pilC1* | GTAGGAGTAGCCTGCCAACC  GCGCCATAGAGTGGGCTAAA | 627 | DE8555f  DE8555r  DE8539f  DE8539r | 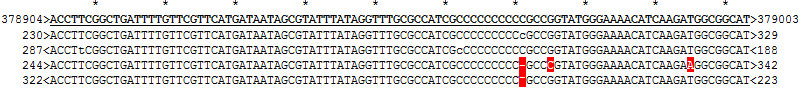 |
| Nd | Nd | C_9_2051444C_10_ | *fetA* | TTCCGATAGATTCCTGCCGC  AGAGTTCGCGCATATCGGTT | 602 | DE8669f  DE8669r  DE8678f  DE8678r | 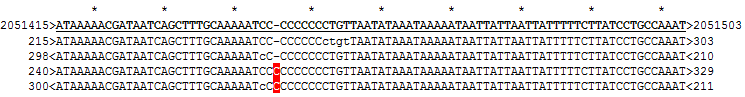 |
| Off | Off | (AGCC)_14_1473315(AGCC)_15_ | *modA12* | AGCATTTCCGTCATCATCAATGG  ACACAAAATACGCCAACCGC | 646 | DE8669f  DE8669r  DE8678f  DE8678r | 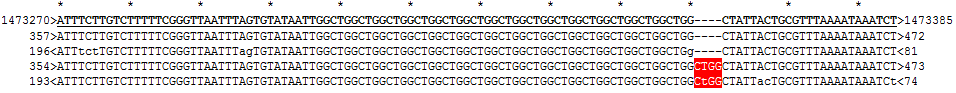 |
| - | - | T1739009A  A1739015G  A1739018G  G1739138A  G1739144C | *tpsS1* | TTGGCGCATGCAGTCATTTC  CCTGAAAGAGCTGGGCAGAA | 612 | DE10444f  DE10444r  DE10445f  DE10554r | 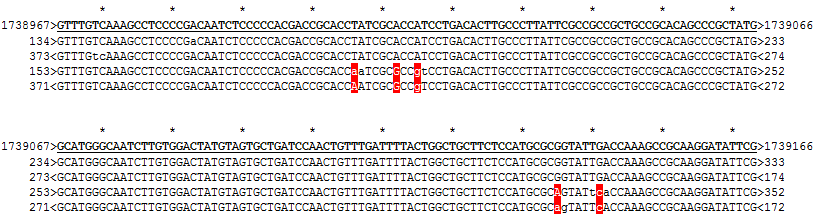 |
| - | - | G16384T  G16419T  C16452T  G16456T  A16481T  G16483T  G16488T  T16489C | *pilE* | AGAGCCTTGAAGCGCAGTC  CAACAACGACGAAGTCACCG | 620 | DE10444f  DE10444r  DE10445f  DE10445r | 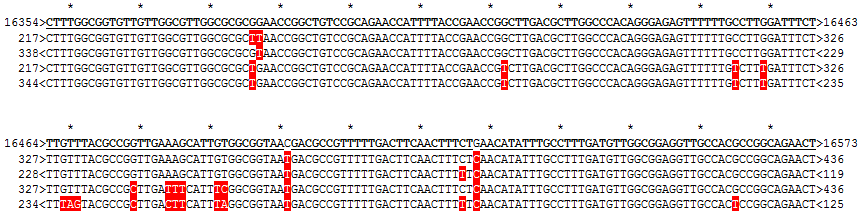 |
| On | Off | G_10_1833654G_9_ | *pilC1* | CCAAAGGGGTTTTCCAACCG  ATTGTTGCGCTTTTCCAGCC | 627 | DE10444f  DE10444r  DE10445f  DE10445r | 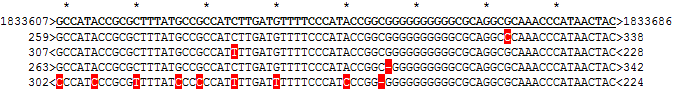 |
| On | Off | G_10_376831G_9_ | *pilC1* | GCATTGCCTTGTGTCTCTGC  CACGAAACACCCTGCGAAAC | 611 | WUE2121f  WUE2121r  WUE2120f  WUE2120r | 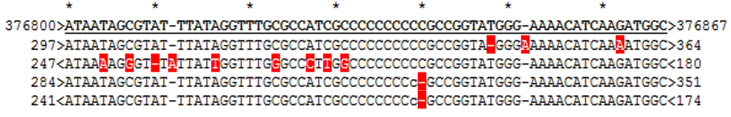 |
| On | off | G_10_386208G_11_ | *pglI* | ATATCCCGAGCCCCGATACA  CGAACGCGGAAGGCATAAAG | 740 | WUE2121f  WUE2121r  WUE2120f  WUE2120r | 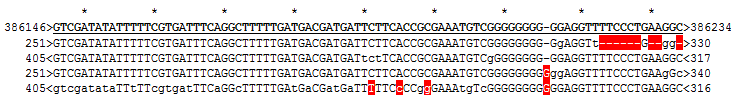 |
| On | on | (GCC)_3_1037014(GCC)_2_ | Phage tail protein | GAAGGCAACACAACTGGCTG  TTCCGATAGATTCCTGCCGC | 567 | WUE2121f  WUE2121r  WUE2120f | 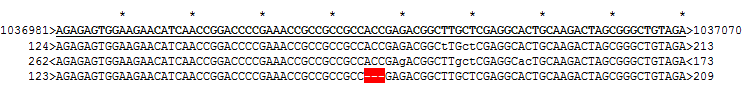 |
| Off | off | (AGCC)_12_1290184(AGCC)_11_ | *modA12* | AACCCAAGCCCTGCTATCAC  GAGATTTTCGGCTGGTGTGC | 618 | WUE2121f  WUE2121r  WUE2120f  WUE2120r | 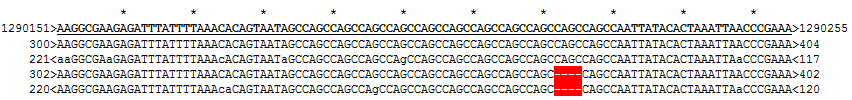 |

**S2 Table.** **Variant validation by Sanger sequencing.**

*The first line in each alignment represents the reference. Each of the following lines represents a Sanger-sequence of an isolate as indicated in the “order” column (f: foreword, r: reverse, blue: throat isolate, red: blood isolate). Differences between the reference (top line) and Sanger-sequences are highlighted by red boxes in the Sanger-sequences.
