# Supplementary material for: Comparative Genome Sequencing Reveals Within-Host Genetic Changes in Neisseria meningitidis during Invasive Disease
Source: PLoS One. 2017 Jan 12;12(1):e0169892. doi: 10.1371/journal.pone.0169892 (PMC5231331; doi:10.1371/journal.pone.0169892)
Supplement: S3 Table — (DOCX) [file pone.0169892.s006.docx]

**S3 Table. Strains and genome sequences used for *in silico* genome comparisons.**

| **Strain** | **Serogoup** | **Sequence type** | **Clonal Complex** | **Geographic origin** | **Year** | **Disease** | **Genome accession** | **CDS** | **SSR in CDS** | **SSR in 5’-UTR** | **Mononucleotide SSR** | **Genome reference** |
| --- | --- | --- | --- | --- | --- | --- | --- | --- | --- | --- | --- | --- |
| α14 | Cnl | 53 | CC53 | Germany | 1999 | Carriage | NC_013016 | 1919 | 213 | 40 | 32 | Schoen *et al.* 2008 (1) |
| 8013 | C | 18 | CC18 | France | 1989 | IMD | NC_017501 | 1912 | 238 | 60 | 41 | Rusniok *et al.* 2009 (2) |
| 53442 | C | 4821 | CC4821 | China | 2003 | IMD | NC_010120 | 1966 | 212 | 50 | 47 | Peng *et al.* 2008 (3) |
| 510612 | A | 7 | CC7 | China | 2014 | IMD | CP007524 | 2107 | 226 | 51 | 53 | Zhang et al. 2014 (4) |
| B6116/77 | B | 10 | CC8 | Iceland | 1977 | IMD | CP007667 | 2086 | 231 | 51 | 50 | Seib *et al.* (2015) (5) |
| DE10444 | Y | 23 | CC23 | Germany | 2005 | IMD | CP012392 | 1993 | 206 | 46 | 38 | This work |
| DE8555 | C | 11 | CC11 | Germany | 2002 | IMD | CP012393 | 2033 | 224 | 49 | 41 | This work |
| DE8669 | B | 42 | CC42 | Germany | 2002 | IMD | CP012391 | 2019 | 205 | 46 | 42 | This work |
| FAM18 | C | 11 | CC11 | USA | 1983 | IMD | NC_008767 | 1949 | 225 | 46 | 42 | Bentley *et al.* 2007 (6) |
| G2136 | B | 8 | CC8 | UK | 1986 | IMD | NC_017513 | 2030 | 230 | 45 | 54 | Budroni *et al.* 2011 (7) |
| H44/76 | B | 32 | CC32 | Norway | 1976 | IMD | NC_017516 | 2076 | 223 | 52 | 44 | Budroni *et al.* 2011 (7) |
| LNP21362 | B | 32 | CC32 | France | 2004 | IMD | CP006869 | 2023 | 237 | 58 | 58 | Institut Pasteur (8) |
| M01-240149 | B | 41 | CC41/44 | UK | 2001 | IMD | CP002421 | 2037 | 210 | 44 | 40 | Budroni *et al.* 2011 (7) |
| M01-240355 | B | 213 | CC213 | UK | 2001 | IMD | NC_017517 | 2061 | 215 | 37 | 33 | Budroni *et al.* 2011 (7) |
| M04-240196 | B | 269 | CC269 | UK | 2004 | IMD | NC_017515 | 2009 | 227 | 49 | 49 | Budroni *et al.* 2011 (7) |
| M0579 | B | 43 | CC41/44 | USA | 1993 | IMD | CP007668 | 2194 | 218 | 44 | 41 | Budroni *et al.* 2011 (7) |
| M10208 | W | 11 | CC11 | Chile | 2014 | IMD | CP009422 | 2039 | 216 | 60 | 48 | University of Pittsburgh (8) |
| M7124 | W | 11 | CC11 | Saud Arabia | 2000 | IMD | CP009419 | 2087 | 215 | 56 | 42 | CDC Atlanta (8) |
| MC58 | B | 74 | CC32 | UK | 1985 | IMD | AE002098 | 2014 | 246 | 50 | 52 | Tettelin *et al.* 2000 (9) |
| NM3682 | /W | 11 | CC11 | UK | 2014 | IMD | CP009420 | 2073 | 216 | 62 | 47 | University of Pittsburgh (8) |
| NM3683 | W | 11 | CC11 | Canada | 1970 | IMD | CP009421 | 2091 | 219 | 66 | 51 | University of Pittsburgh (8) |
| NM3686 | W | 11 | CC11 | Brazil | 2014 | IMD | CP009418 | 2111 | 219 | 63 | 51 | University of Pittsburgh (8) |
| NZ-05/33 | B | 42 | CC41/44 | New Zealand | 2005 | IMD | NC_017518 | 1995 | 211 | 45 | 42 | Budroni *et al.* 2011 (7) |
| WUE2121 | C | 11 | CC11 | Germany | 1997 | IMD | CP012394 | 2039 | 226 | 46 | 43 | This work |
| WUE2594 | A | 5 | CC5 | Germany | 1991 | IMD | NC_017512 | 1941 | 213 | 47 | 40 | Schoen *et al.* 2011 (10) |
| Z2491 | A | 4 | CC4 | Gambia | 1983 | IMD | NC_003116 | 1979 | 213 | 35 | 38 | Parkhill *et al.* 2000 (11) |
| α710 | B | 136 | CC41/44 | Germany | 2000 | Carriage | NC_017505 | 1975 | 239 | 37 | 48 | Joseph *et al.* 2010 (12) |

1. Schoen C*, et al.* (2008) Whole-genome comparison of disease and carriage strains provides insights into virulence evolution in Neisseria meningitidis. *Proceedings of the National Academy of Sciences of the United States of America* 105(9):3473-3478.

2. Rusniok C*, et al.* (2009) NeMeSys: a biological resource for narrowing the gap between sequence and function in the human pathogen Neisseria meningitidis. *Genome biology* 10(10):R110.

3. Peng J*, et al.* (2008) Characterization of ST-4821 complex, a unique Neisseria meningitidis clone. *Genomics* 91(1):78-87.

4. Zhang Y, *et al.* (2014) Complete Genome Sequence of *Neisseria meningitidis* Serogroup A Strain NMA510612, Isolated from a Patient with Bacterial Meningitis in China. *Genome Announc* 2(3): e00360-14.

5. Seib KL, *et al.* (2015) Specificity of the ModA11, ModA12 and ModD1 epigenetic regulator N6-adenine DNA methyltransferases of *Neisseria meningitidis*. *Nucleic Acids Res* 43:4150-4162.

6. Bentley SD*, et al.* (2007) Meningococcal genetic variation mechanisms viewed through comparative analysis of serogroup C strain FAM18. *PLoS genetics* 3(2):e23.

7. Budroni S*, et al.* (2011) Neisseria meningitidis is structured in clades associated with restriction modification systems that modulate homologous recombination. *Proceedings of the National Academy of Sciences of the United States of America* 108(11):4494-4499.

8. Information taken from the Neisseria PubMLST website (pubmlst.org/neisseria/)

9. Tettelin H*, et al.* (2000) Complete genome sequence of Neisseria meningitidis serogroup B strain MC58. *Science (New York, N.Y.)* 287(5459):1809-1815.

10. Schoen C*, et al.* (2011) Whole-genome sequence of the transformable Neisseria meningitidis serogroup A strain WUE2594. *Journal of bacteriology* 193(8):2064-2065.

11. Parkhill J*, et al.* (2000) Complete DNA sequence of a serogroup A strain of Neisseria meningitidis Z2491. *Nature* 404(6777):502-506.

12. Joseph B*, et al.* (2010) Comparative genome biology of a serogroup B carriage and disease strain supports a polygenic nature of meningococcal virulence. *Journal of bacteriology* 192(20):5363-5377.
