# Supplementary material for: Comparative Genome Sequencing Reveals Within-Host Genetic Changes in Neisseria meningitidis during Invasive Disease
Source: PLoS One. 2017 Jan 12;12(1):e0169892. doi: 10.1371/journal.pone.0169892 (PMC5231331; doi:10.1371/journal.pone.0169892)
Supplement: S4 Table — (DOCX) [file pone.0169892.s007.docx]

S4 Table. SSR loci encoding proteins of the COG functional category M (cell wall/membrane/envelope biogenesis).

| CDS | Gene | Copy Number | Repeat sequence | Repeat location | Type |
| --- | --- | --- | --- | --- | --- |
| Capsule biosynthesis |  |  |  |  |  |
| NMC0057 | *ctrC* | 4.3 | TGT | CDS | variable |
| NMAA_1902 | *ctrC* | 4.3 | GAT | CDS | variable |
| NMC0058 | *ctrD* | 4.3 | TGT | Promoter | variable |
| NMV_0071 | *siaA* | 3.3 | TACTTATA | Promoter | variable |
| LPS biosynthesis |  |  |  |  |  |
| DE8669_142 | *galE* | 4 | CAG | Promoter | variable |
| NMB0014 | *kdtA* | 10 | G | CDS | variable |
| NMB1929 | *lgtA* | 14 | G | CDS | variable |
| NMBB_2204 | *lgtC* | 10 | G | CDS | variable |
| NMC0191 | *lpxB* | 5 | CG | CDS | core |
| NMC0001 | *lpxC* | 6.5 | GC | CDS | core |
| NMB2156 | *rfaC* | 3.3 | AATA | Promoter | variable |
| NMC1456 | *rfaF* | 4 | TG | CDS | variable |
| NMC0401 | *rfaG* | 22 | C | CDS | variable |
| NMCC_0071 | *rfbB* | 5.5 | GC | CDS | variable |
| Peptidoglycan biosynthesis | | | | | |
| B6116_00814 | *mrcA* | 8 | G | CDS | variable |
| NMC1749 | *murF* | 3.3 | GCGG | CDS | variable |
| NMC1742 | *murG* | 5.5 | CG | CDS | variable |
| NMBG2136_0420 | *pbp3* | 8 | C | CDS | variable |
| NMO_1122 | *mltB* | 3 | GCCC | CDS | variable |
| NMC0010 | *gna33* | 4 | TG | CDS | variable |
| NMC1694 | *amiC* | 7 | C | CDS | variable |
| Tfp biosynthesis |  |  |  |  |  |
| NMB0218 | *pglA* | 11 | G | CDS | variable |
| NMC0568 | *pglE* | 7 | G | Promoter | variable |
|  |  | 5 | AT | CDS |  |
|  |  | 30.7 | AAACAAC | CDS |  |
| Autotransporter |  |  |  |  |  |
| NMC1454 | *autB* | 7.3 | AGCA | Promoter | variable |
| NMC1772 ^(1)^ | *nadA* | 4.3  2 | GCG  GCGGCGG | CDS  CDS | variable |
| NMB1998 | *iga* | 9 | C | CDS | variable |
|  |  | 4.5 | CA | CDS |  |
| NMC1959 | *iga2* | 3 | GGCA | CDS | variable |
| Outer membrane proteins and adhesins | | | | | |
| NMBG2136_0032 | *glmS* | 9 | T | Promoter | variable |
| NMB1053^(2)^ | *opc* | 12 | G | Promoter | variable |
| NMB1716 | *mtrC* | 6 | GC | CDS | variable |
| NMC0173 | *omp85* | 4 | AC | CDS | variable |
| NMC1403 | *opaA* | 11.8 | TCTTC | CDS | variable |
| NMC1551 | *opaB* | 6.8 | CTTCT | CDS | variable |
| NMC1719 | *opaC* | 11.8 | CTTCT | CDS | variable |
| NMC0903 | *opaD* | 8.8 | TCTTC | CDS | variable |
| NMC1364 | *porA* | 7 | G | Promoter | variable |
| NMO_1728 | *vapA* | 17 | AAGC | CDS | variable |
| Unspecified |  |  |  |  |  |
| DE10444_1921 | NA | 7 | C | CDS | variable |
| B6116_00799 | NA | 14 | C | CDS | variable |
| B6116_01518 | NA | 9 | A | Promoter | variable |
| NMA0615 | NA | 4 | AT | CDS | variable |
| NMA0640 | NA | 7 | C | CDS | variable |
| NMAA_0342 | NA | 3.3 | CAGG | CDS | variable |
| NMB1693 | NA | 5.5 | CA | CDS | variable |
| NMBB_0393 | NA | 6.5 | GC | CDS | variable |
| NMBG2136_0982 | NA | 4 | CA | CDS | variable |
| NMBH4476_0091 | NA | 9 | T | Promoter | variable |
| NMBM04240196_1864 | NA | 7 | G | CDS | variable |
| NMBNZ0533_0977 | NA | 7.8 | TCTTC | Promoter | variable |
| NMC1156 | NA | 9 | G | CDS | variable |
| NMC1208 | NA | 4.5 | GT | CDS | variable |
| NMC1401 | NA | 4 | GAG | CDS | variable |
| NMC1926 | NA | 3.8 | CGCC | CDS | core |
| NMC2011 | NA | 14 | C | CDS | variable |
| NMO_0975 | *lgt* | 10 | C | Promoter | variable |

^(1)^ Although both repeat types did not meet the inclusion criteria used for the computational analysis *nadA* was included in this table as it represents an important adhesin and vaccine candidate.

^(2)^ Although *opc* does not belong to COG class M it is a major adhesin in *N. meningitidis* and was therefore included in this table.
