# Supplementary material for: Comparative Genome Sequencing Reveals Within-Host Genetic Changes in Neisseria meningitidis during Invasive Disease
Source: PLoS One. 2017 Jan 12;12(1):e0169892. doi: 10.1371/journal.pone.0169892 (PMC5231331; doi:10.1371/journal.pone.0169892)
Supplement: S5 Table — (DOCX) [file pone.0169892.s008.docx]

S5 Table: Repeat tract length variation of *fetA* and *pilC1* during five in vitro passages of strain WUE2121. The reference tract lengths for *fetA* and *pilC1* are highlighted.

| **Tract length** | ***fetA*** | | | | ***pilC1*** | | | |
| --- | --- | --- | --- | --- | --- | --- | --- | --- |
|  | **Single-colony transfer from third streak area** | | **Transfer from the confluent third streak area** | | **Single-colony transfer from third streak area** | | **Transfer from the confluent third streak area** | |
|  | Inoculum | Passaged | Inoculum | Passaged | Inoculum | Passaged | Inoculum | Passaged |
| 9 | 0 | 0 | ND | 0 | 1 | 0 | ND | 6 |
| 10 | 1 | 1 | ND | 0 | **8** | **10** | **ND** | **4** |
| 11 | **1** | **5** | **ND** | **2** | 0 | 0 | ND | 0 |
| 12 | 2 | 1 | ND | 5 | 0 | 0 | ND | 0 |
| 13 | 3 | 2 | ND | 3 | 0 | 0 | ND | 0 |
| 14 | 0 | 1 | ND | 0 | 0 | 0 | ND | 0 |
| Coeffcient of variation | 10% | 11% | ND | 6% | 3% | 0% | ND | 5% |
